# Nasopharyngeal Bacterial Microbiota Composition and SARS-CoV-2 IgG Antibody Maintenance in Asymptomatic/Paucisymptomatic Subjects

**DOI:** 10.3389/fcimb.2022.882302

**Published:** 2022-07-06

**Authors:** Luca Ferrari, Chiara Favero, Giulia Solazzo, Jacopo Mariani, Anna Luganini, Monica Ferraroni, Emanuele Montomoli, Gregorio Paolo Milani, Valentina Bollati

**Affiliations:** ^1^ EPIGET Lab, Department of Clinical Sciences and Community Health, Università degli Studi di Mila-no, Milan, Italy; ^2^ Department of Preventive Medicine, Fondazione IRCCS Ca’ Granda Ospedale Maggiore Policlinico, Milan, Italy; ^3^ Laboratory of Microbiology and Virology, Department of Life Sciences and Systems Biology, Università degli Studi di Torino, Turin, Italy; ^4^ Branch of Medical Statistics, Biometry, and Epidemiology "G. A. Maccacaro", Department of Clinical Sciences and Community Health, Università degli Studi di Milano, Milan, Italy; ^5^ Department of Molecular and Developmental Medicine, Università degli Studi di Siena, Siena, Italy; ^6^ Department of Clinical Sciences and Community Health, Università degli Studi di Milano, Milan, Italy; ^7^ Pediatric Unit, Fondazione IRCCS Ca’ Granda Ospedale Maggiore Policlinico, Milan, Italy

**Keywords:** UNICORN, SARS-CoV-2, nasopharyngeal bacterial microbiota, immunoglobulins, asymptomatic carriers

## Abstract

The severe acute respiratory syndrome coronavirus 2 (SARS-CoV-2) causes the coronavirus disease 2019 (COVID-19), ranging from asymptomatic conditions to severe/fatal lung injury and multi-organ failure. Growing evidence shows that the nasopharyngeal microbiota composition may predict the severity of respiratory infections and may play a role in the protection from viral entry and the regulation of the immune response to the infection. In the present study, we have characterized the nasopharyngeal bacterial microbiota (BNM) composition and have performed factor analysis in a group of 54 asymptomatic/paucisymptomatic subjects who tested positive for nasopharyngeal swab SARS-CoV-2 RNA and/or showed anti-RBD-IgG positive serology at the enrolment. We investigated whether BNM was associated with SARS-CoV-2 RNA positivity and serum anti-RBD-IgG antibody development/maintenance 20–28 weeks after the enrolment. Shannon’s entropy α-diversity index [odds ratio (OR) = 5.75, p = 0.0107] and the BNM Factor1 (OR = 2.64, p = 0.0370) were positively associated with serum anti-RBD-IgG antibody maintenance. The present results suggest that BNM composition may influence the immunological memory against SARS-CoV-2 infections. To the best of our knowledge, this is the first study investigating the link between BNM and specific IgG antibody maintenance. Further studies are needed to unveil the mechanisms through which the BNM influences the adaptive immune response against viral infections.

## Introduction

The severe acute respiratory syndrome coronavirus 2 (SARS-CoV-2) has been infecting millions of people and causing more than five million deaths worldwide since the end of 2019 (Wu and McGoogan, 2020; WHO, 2021). The SARS-CoV-2 virus infection causes the coronavirus disease 2019 (COVID-19), ranging in presentation from asymptomatic to severe lung injury and multi-organ failure, eventually leading to death (Berlin et al., 2020; Gandhi et al., 2020; Vicenzi et al., 2020). The host features influence both the severity and outcomes of SARS-CoV-2 infection (Lauer et al., 2020; Sun et al., 2020), and the local and systemic immune responses play a key role in the reaction to the viral threat especially in the first stage of disease (Tay et al., 2020). Most of the infected individuals experience asymptomatic to mild symptomatic conditions, but only some of them develop antibodies (Milani et al., 2020a; Milani et al., 2020b).

SARS-CoV-2 binds to the host cells through the interaction between the receptor-binding domain (RBD), present in the viral spike (S) glycoprotein, and the angiotensin-converting enzyme 2 (ACE2) on host cells (Hoffmann et al., 2020). Most SARS-CoV-2-infected individuals produce S- and RBD-specific antibodies during the first 2 weeks of the primary response, and RBD-specific antibodies can neutralize the virus *in vitro* and *in vivo* (Rodda et al., 2021).

SARS-CoV-2 virus penetrates the host through the upper airways, and the nasal barrier is the first defensive line to limit infection (Tay et al., 2020). In addition to the epithelial layer and the local immune system, the upper airways harbor a community of microorganisms, the nasopharyngeal microbiota, which is pivotal in maintaining mucosal homeostasis and in the resistance to infections (Man et al., 2017). Growing evidence shows that the nasopharyngeal microbiota composition may help to predict the severity of respiratory infections (de Steenhuijsen Piters et al., 2015; Kumpitsch et al., 2019; Man et al., 2019). However, the role of the upper airway microbiota in COVID-19 is far from being understood and likely goes beyond protection from viral entry to include the regulation of the immune response to the infection (Di Stadio et al., 2020).

The present study was aimed at characterizing the nasopharyngeal bacterial microbiota (BNM) by 16S rRNA gene sequencing in a group of 54 asymptomatic/paucisymptomatic subjects who tested positive for nasopharyngeal swab SARS-CoV-2 RNA and/or showed positive serology for anti-RBD-IgG at the enrolment. We investigated whether the composition of the BNM collected at the enrolment was associated with serum anti-RBD-IgG development and maintenance after 20–28 weeks. This study was part of the UNICORN (“UNIversity against CORoNavirus”) project, which was conducted among the personnel of the University of Milan (Milani et al., 2020a; Milani et al., 2020b, Milani et al., 2021).

## Materials and Methods

The investigated subjects are a subset of the UNICORN study. The enrolment criteria and procedures were previously described (Milani et al., 2021). Briefly, all the participants in the study were volunteers working at the University of Milan. In this specific study, antibiotic consumption up to 1 month before the enrolment was considered an exclusion criterion. Other excluding criteria were fever, any symptoms of flu-like infections or dyspnea at the time of the recruitment or during the preceding 14 days, prolonged and close contact with any subjects positive for SARS-CoV-2, or symptoms suggestive of infection during the previous 14 days. The study was approved by the ethics committee of the University of Milan (approval number 17/20; approval date March 6, 2020; amendment date November 17, 2020) and conducted following the Declaration of Helsinki. All participants signed an informed consent form.

This investigation includes 54 subjects selected among those who tested positive for either SARS-CoV-2 RNA nasopharyngeal swab or serum anti-RBD IgG antibodies in the UNICORN study population. The present study includes the subjects who donated the nasal swab within 3 months from the beginning of the pandemic in Italy (during the first wave of SARS-CoV-2, from March to June 2020) and whose DNA yield and quality were acceptable to perform the 16S sequencing (yield > 100 ng; purity 260/280 ratio > 1.8; 260/230 ratio 1.8–2.1).

### Nasopharyngeal Sample Collection and SARS-CoV-2 RNA Detection

Nasopharyngeal swabs were collected from each participant, viral RNA was extracted, and SARS-CoV-2 RNA was detected as previously detailed (Milani et al., 2021). Briefly, RNA was isolated from swabs by using the QIAamp Viral RNA Mini Kit (Qiagen, Hilden, Germany), according to the manufacturer’s instructions. SARS-CoV-2 RNA detection was performed by using the multiplex real-time quantitative PCR test TaqPath COVID-19 CE-IVD RT-PCR Kit, Thermo Fisher Scientific (Waltham, MA, USA) following the manufacturer’s instructions. In each extracted sample, 10 µl of internal control RNA (i.e., MS2 Phage) and an RNA carrier were added before being stored at −80°C. In the PCR, specific probes were annealed to three specific SARS-CoV-2 sequences: 1) ORF1ab with reporter dye FAM; 2) N protein (nucleocapsid) with reporter dye VIC; and 3) S protein with reporter dye ABY. The MS2 internal control-specific probe (labeled with the JUN dye) was included to verify the efficacy of the sample preparation. After RNA was reverse transcribed into cDNA, samples were amplified using the QuantStudio 12K Flex Real-Time PCR Instruments (Thermo Fisher). The data analysis was performed using the “Design and Analysis Software” (V.2.3.3, Thermo Fisher) setting “Automatic Threshold.” The reaction was considered only if the MS2 cycle threshold (Ct) ≤38. If any two of the three SARS-CoV-2 genes were positive (Ct ≤38), the sample was classified as positive; if only one of the assays was positive, the test was repeated. If after repetition the sample tested positive again, the sample was classified as positive for SARS-CoV-2 RNA. If all three of the assays were negative (Ct = undetermined), the subject was classified as negative.

### 16S rRNA Gene Sequencing

DNA from nasopharyngeal swabs was extracted by using QIAamp^®^ UCP Pathogen Mini (Qiagen, Hilden, Germany) following the manufacturer’s guidelines. The extracted DNA was stored at −20°C and later shipped to the sequencing service facility Personal Genomics Srl (Verona, Italy) for qualitative and quantitative checks, PCR amplification, and second-generation sequencing analysis. Four extraction- and PCR-negative controls were included in the procedure, but library preparation for these control samples failed. Libraries were obtained by following the Illumina 16S Metagenomic Sequencing Library Preparation (Illumina, San Diego, CA, USA). The bacterial microbiome was investigated by amplicon sequencing analysis of the 16S rRNA gene hypervariable regions V3–V4, amplified with the following oligonucleotides: Pro341F (5′-CCTACGGGNBGCASCAG-3′) and Pro805R (5′-GACTACNVGGGTATCTAATCC-3′). Sequencing was performed with the Illumina MiSeq platform (Illumina) by using a paired-end library of 300-bp insert size.

### Upstream Analyses and Operational Taxonomic Unit Clustering

Raw read quality and statistics were checked using FastQC v0.11.2 and then imported into QIIME2 v2020.6 (Bolyen et al., 2019) software for the following analysis. Primer sequences were removed from each read with cutadapt plugin using the trim-paired method to improve database read matching. The trimmed files were then joined using Vsearch’s merge_pairs function with a minimum overlap length of forward and reverse reads of 80 bp, to cover the 16S V3–V4 region (Rognes et al., 2016). Then, joined reads underwent a quality filtering process to exclude from further analysis those reads with a quality value less than a PHRED score of 20 on a base-slide window of 3 nucleotides. The retained joined reads were then grouped into high-resolution amplicon sequence variants (ASVs) using the Deblur denoiser plugin with an arbitrary minimum length of 400 bp to be retained (Amir et al., 2017). Taxonomic assignment was done through the skylearn-classifier against the SILVA v132_99_16S database, which had been modified to contain only the V3–V4 16S fragments to improve read matching. Mafft-fast-tree method and default setting suggested in the QIIME2 pipeline were applied to align the sequences and to generate rooted and unrooted trees for phylogenetic analysis.

### Downstream Analysis

Downstream analyses were carried out using QIIME2 v2020.62 analyzing the above-described ASV or feature table. Taxonomic values within each sample and group were assigned to each ASV from the phylum to the genus level. ASVs that failed genus attribution were tagged as “Unassigned” followed by the specific family label. Before diversity analysis, all samples were rarefied to 10,000 sequences with a seed of 10 in order to avoid the influence of different sequencing depths, as this number of sequences was the minimum identified in the ASVs table. α-Diversity richness, evenness, and genetic distance were calculated using observed ASVs, Shannon, and Faith’s phylogenetic diversity (Faith’s PD) indices.

### Blood Collection and Serum Anti-RBD-IgG Detection

Blood samples were collected in ethylenediamine tetra-acetic acid (EDTA) tubes and processed within 2 h of the phlebotomy. The detection of specific anti-RBD-IgG antibodies was performed by an ELISA approach that was previously described (Mazzini et al., 2021; Milani et al., 2021). Briefly, for the detection of anti-RBD IgG, ELISA plates were coated with purified recombinant spike-RBD HEK-derived protein (Sino Biological, Beijing, China). Serum samples were heat-inactivated at 56°C for 1 h and diluted at 1:100 in Tris-buffered saline (TBS)–0.05% Tween 20 5%. Each serum dilution measuring 100 µl was added to the coated plates with specific antibodies and incubated for 1 h at 37°C. Then, 100 µl/well of Goat anti-Human IgG-Fc horseradish peroxidase (HRP)-conjugated antibody (dilution 1:100,000; Bethyl Laboratories, Montgomery, TX, USA) was added. After incubation at 37°C for 30 min, plates were washed and 100 µl/well of 3,3′,5,5′-tetramethylbenzidine substrate (Bethyl Laboratories) was added in the dark at room temperature for 20 min. After stopping the reaction with 100 µl of ELISA stop solution (Bethyl Laboratories), plates were read at 450 nm, with a cutoff value established as three times the average optical density (OD) values from blank wells (background—no addition of analyte). Borderline samples were defined where one replicate was under the cutoff and the other was above. Sensitivity was reported to be 85.7% and specificity 98.1%.

### Statistical Analysis

Descriptive statistics were performed on all variables. Quantitative data were expressed as mean ± SD or as median [first quartile–third quartile] if not normally distributed. Categorical data were presented as frequencies and percentages. Continuous variables were tested for normality and linearity. Factor analysis was applied to reduce a large dimension of microbiome data to a smaller number of latent independent factors to predict microbiome composition at the genus level (**Supplementary Figure S1**). A set of 47 genera, excluding *a priori* two genera (i.e., “:” and “uncultured”), were selected because they did not provide any interpretable results. Next, the correlation matrix of the log-transformed variables was analyzed. Since *Sphingomonas* and *Streptococcus* genera did not correlate (p-value >0.05) with any other genera and correlation coefficients were less than |0.25|, they were not included in the factor analysis. Whether the correlation matrix of the log-transformed relative abundances of 45 genera was factorable was evaluated by visual inspection of the matrix as well as statistical procedures, including Bartlett’s test of sphericity, overall [Kaiser–Meyer–Olkin (KMO)], and individual measures of sampling adequacy (**Table 1**). An overall KMO ≤ 0.50 for the factor analysis and genera with a measure of sampling adequacy <0.30 (Rajalahti and Kvalheim, 2011) were considered unacceptable. Thus, 20 genera were excluded, and the method assumption on the correlation matrix was verified again considering the remaining 25 genera. The new correlation matrix was factorable, but six genera (*Staphylococcus*, *Campylobacter*, *Clostridium senso stricto 10*, *Moraxella*, *Escherichia-Shigella*, and *Corynebacterium 1*) were excluded because of their low communality; i.e., they explained less than 15% of variance each. In the last correlation matrix, all the assumptions were satisfied, and factor analysis was applied to obtain the microbiome patterns.

**Table 1:** Factorability of the correlation matrix of the log-transformed genera: Bartlett’s test of sphericity and measures of sampling adequacy.

	from correlation matrix N=45	from correlation matrix N=25	from correlation matrix N=19
**Bartlett's test of sphericity:**	p-value <0.0001	p-value <0.0001	p-value <0.0001
**Kaiser-Meyer-Olkin statistic - Overall measure of sampling adequacy:**	0.36	0.69	0.70
**Individual measures of sampling adequacy:**			
< 0.30	*Paracoccus, Mesorhizobium, Neisseria, Lawsonella, Citrobacter, Ralstonia, Carnobacterium, Dolosigranulum, Micrococcus, Peptoniphilus, Anaerococcus, Acinetobacter, Finegoldia, Geobacillus, Enhydrobacter, Deinococcus, Serratia, Labrys, Gemella, Thermosinus*	–	–
0.30 - 0.40	*Afipia, Staphylococcus, Escherichia Shigella, Caldicellulosiruptor, Vibriomonas, Corynebacterium 1, Sediminbacterium*	*Staphylococcus*	–
0.40 - 0.50	*Thermus, Clostridium senso stricto 10, Cutibacterium, Bacillus, Tepidiphilus, Bradyrhizobium, Moraxella, Campylobacter*	*Afipia, Vibriomonas, Campylobacter*	*Afipia, Vibriomonas*
0.50 - 0.60	*Thermoanaerobacter, Pseudomonas, Aeromonas, Enterococcus*	*Bradyrhizobium, Sediminbacterium, Pseudomonas*	*Bradyrhizobium, Pseudomonas, Sediminbacterium*
0.60 - 0.70	*Gulbenkiania, Thermoanaerobacterium, Tumebacillus, Fervidobacterium, Comamonas*	*Thermus, Thermoanaerobacterium, Caldicellulosiruptor, Clostridium senso stricto 10, Enterococcus*	*Thermus, Thermoanaerobacterium, Caldicellulosiruptor, Enterococcus*
0.70 - 0.80	*Burkholderia Caballeronia Parabulkholderia*	*Cutibacterium, Escherichia Shigella, Tepidiphilus, Moraxella, Thermoanaerobacter, Gulbenkiania, Tumebacillus, Aeromonas, Corynebacterium 1*	*Thermoanaerobacter, Tepidiphilus, Gulbenkiania, Tumebacillus*
0.80 - 0.90	*-*	*Comamonas, Bacillus, Fervidobacterium, Burkholderia Caballeronia Parabulkholderia*	*Aeromonas, Enterococcus, Bacillus, Thermosinus, Thermoanaerobacter, Comamonas, Gulbenkiania, Burkholderia Caballeronia Parabulkholderia*
≥ 0.90	-	-	-

Overall and individual measures of sampling adequacy range between 0 and 1, with values > 0.50 indicating an acceptable size.

Exploratory principal component factor analysis was performed on the correlation matrix of nineteen selected genera to identify a smaller set of uncorrelated underlying factors. The number of factors to be included in the analysis was chosen considering the following criteria: factor eigenvalues > 1, scree-plot construction, and factor interpretability (Härdle and Simar, 2012). A varimax rotation to the factor-loading matrix was applied to obtain a simpler loadings structure and improve the interpretation. Genera with an absolute rotated factor loading ≥ 0.63 on a given factor were used to name the factor and are indicated as “dominant genera” hereafter (Gudgeon et al., 1994). Factor scores, calculated for each subject and each pattern, indicated how consistent was each participant’s microbiome with the identified pattern. To confirm both reproducibility and stability of the identified independent factors, additional exploratory factor analyses were carried out to derive factor scores from all genera (n = 45) and 25 genera with KMO ≥ 0.30. Given the reassuring and consistent results from this check, all the subsequent analyses on the factor scores derived from the subset of 19 genera were carried out. To assess the reliability of microbiome patterns and internal consistency of genera that load more than |0.40| on any factor, Cronbach’s coefficient alpha for each factor and coefficient alpha when the item was deleted were calculated. Next, two different outcomes were focused on. First, whether the microbiome influenced the probability of developing IgG antibodies was verified at both the baseline (i.e., enrolment T1) and the follow-up (T2). Second, whether the microbiome composition modified the probability to maintain anti-RBD IgG antibodies at the T2 (i.e., 20–28 weeks after enrolment) in subjects with IgG+ at the T1 was investigated.

Multiple logistic regression models were applied to estimate the odds ratios (ORs), and their 95% CI for each microbiome pattern was estimated with factor analysis, α-diversity indices, and relative abundance for each taxon at the phylum and genus levels. One model was fitted for each microbiome pattern. All multivariable models were adjusted for age, gender, smoking habit (yes, no, and former), lifestyle (active and sedentary), and the month of enrolment. Due to the high number of comparisons, multiple comparison correction methods based on the Benjamini–Hochberg false discovery rate (FDR) were applied to calculate the FDR p-value. In the second outcome, the models were adjusted also for SARS-CoV-2 RNA detection at the T1 (positive and negative).

To improve the interpretability of microbiome patterns significantly associated with anti-RBD IgG measured at the T2, a score adding the relative abundance of the overall four dominant genera (i.e., *Enterococcus*, *Pseudomonas*, *Bacillus*, and *Burkholderia Caballeronia Paraburkholderia*) was created in the so-called Factor1. A receiver operating characteristic (ROC) curve was generated to evaluate the diagnostic ability of the microbiome score to distinguish between participants maintaining or non-maintaining IgG at T2. The optimum threshold was selected by Youden’s index as the one that maximized sensitivity (SE) + specificity (SP) − 1. The area under the ROC curve (AUC) and the corresponding 95% CI, SE, SP, and threshold were reported. Statistical analyses and graphs were performed with SAS software (version 9.4; SAS Institute Inc., Cary, NC, USA) and R software (version 4.1.2; Foundation for Statistical Computing, Vienna, Austria).

## Results

### Study Population

The study population was composed of 54 asymptomatic/paucisymptomatic subjects who tested positive for nasopharyngeal swab SARS-CoV-2 RNA and/or showed anti-RBD-IgG antibodies for SARS-CoV-2 at the enrolment (defined as T1). At the T1, 19 out of 54 subjects presented positive nasopharyngeal swab for SARS-CoV-2, while 35 tested positive only for serology of anti-RBD-IgG antibodies. Thus, 6 subjects were positive for both the nasopharyngeal swab and serology at the T1 (**Supplementary Table S1**). At the T2, occurring approximately 20–28 weeks after the T1, 32 out of 41 individuals with positive serology at the T1 (i.e., 35 IgG-positive individuals + 6 swab- and IgG-positive individuals) maintained positive serology. All the participants in the study were employed at the University of Milan, Italy, at the time of the enrollment. Subjects who tested positive for SARS-CoV-2 RNA nasopharyngeal swab were completely asymptomatic at enrolment, while subjects who tested positive for serum anti-RBD IgG antibodies reported completely no symptoms (40.7%), or mild-to-moderate symptoms (51.9% at least one episode of upper airway infections; 20.4% with at least one episode of lower airway infections; 44.4% with at least one episode of fever), which occurred from October 2019 to 14 days before the enrolment (none of them with a previous certified COVID-19 diagnosis). The characteristics of the study population are reported in **Table 2**.

**Table 2:** Characteristics of the study participants.

	All subjects N = 54
**Age,** years mean ± SD	45 ± 12.0
**Gender,** N (%)	
Male	28 (51.9)
Female	26 (48.1)
**BMI,** kg/m^2^, mean ± SD	23.8 ± 4.1
**Smoking,** N (%)	
Never	38 (70.3)
Former	9 (16.7)
Current	7 (13.0)
**Education,** N (%)	
Junior high school	1 (1.9)
High school	10 (18.5)
University	10 (18.5)
Above university	33 (61.1)
**Means of transport to and from work**, N (%)	
Private means of transport	28 (53.9)
Public means of transport	17 (32.7)
Both	7 (13.4)
**Time to and from work**, N (%)	
<1 h	43 (82.7)
1–2 h	9 (17.3)
**Lifestyle,** N (%)	
Sedentary	14 (26.0)
Active	40 (74.0)
**Travels (from October 2019),** N (%)	
Europe (at least one)	21 (38.9)
America (at least one)	6 (11.5)
Oceania (at least one)	0 (0.0)
Asia (at least one)	3 (5.8)
Africa (at least one)	1 (1.9)
**Flu vaccine,** N (%)	
Yes	10 (18.5)
** From October 2019**	
** Upper airway infections,** N (%)	
Yes	28 (51.9)
** Lower airway infections,** N (%)	
Yes	11 (20.4)
** Fever,** N (%)	
Yes	24 (44.4)
** At least one of symptoms,** N (%)	
Yes	32 (59.3)

Continuous variables are expressed as mean ± SD; discrete variables are expressed as counts (%).

BMI, body mass index.

### Nasopharyngeal Bacterial Microbiota Composition and α-Diversity

**Figure 1:**
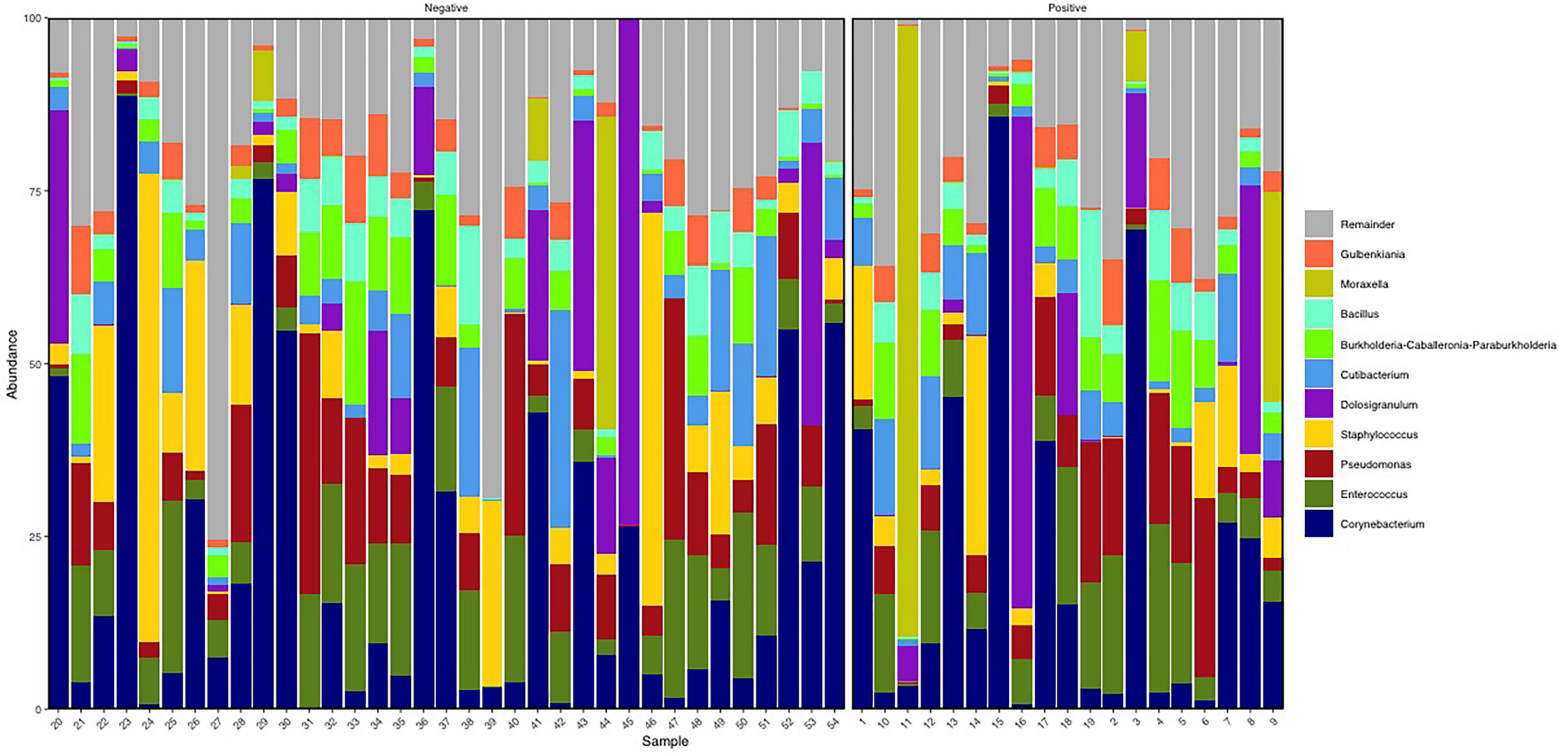
Descriptive nasopharyngeal bacterial microbiota (BNM) genus-profile composition in the two groups SARS-CoV-2 RNA negative (i.e., negative, N = 35) and SARS-CoV-2 RNA positive (i.e., positive, N = 19). Here the top 10 most abundant genera are represented. Figure generated by R software (version 4.1.2 https://www.r-project.org/).

Considering the entire study population, the BNM was dominated by Actinobacteria (relative abundance mean 30.6% (SD ± 24.36%), Firmicutes (36.98% ± 17.6%), and Proteobacteria (30.56% ± 21.28%) phyla (**Supplementary Table S2**). Of the 47 genera detected, the most represented in the study population were *Corynebacterium* (21.95% ± 24.4%), *Enterococcus* (9.78% ± 7.51%), *Staphylococcus* (8.15% ± 13.44%), *Dolosigranulum* (8.14% ± 1.65%), *Pseudomonas* (9.23% ± 8.91%), *Cutibacterium* (6% ± 6.52%), *Burkholderia Caballeronia Paraburkholderia* (5.24% ± 4.66%), *Bacillus* (4.19% ± 3.67%), *Moraxella* (3.53% ± 13.94%), and *Gulbenkiania* (3.35% ± 3.07%) (**Figure 1**; **Supplementary Table S3**). BNM compositional diversity (α-diversity) was calculated for each sample in the study. The richness and phylogenetic diversity evaluated in terms of ASVs showed a mean of 36.85 ( ± 8.15), while the Faith_PD index mean was 3.02 ( ± 0.58). Shannon index, which combines estimates of richness and evenness within the samples, had a mean of 3.42 ( ± 0.90). After univariate analysis, among the 47 genera identified, only *Vibrionimonas* median relative abundance was different in the 19 subjects who were positive for SARS-CoV-2 RNA, compared to the 35 who were negative (SARS-CoV-2 RNA positive, 0.44%; SARS-CoV-2 RNA negative, 0.04%, p-value = 0.02), and no differences were observed for α-diversity indices (**Supplementary Table S4**).

In addition, we performed 16S sequencing in a group of 18 healthy negative control subjects who tested negative for both SARS-CoV-2 RNA and anti-RBD SARS-CoV-2 IgG at the T1, were negative for anti-RBD SARS-CoV-2 IgG at T2, and reported no symptoms attributable to SARS-CoV-2 infection. However, as not all asymptomatic subjects with positive SARS-CoV-2 RNA develop IgG (Milani et al., 2020a), we considered that attributing the negative control status (i.e., assuming no contact with the virus) on the basis of the result of the IgG analysis was not adequate. We thus decided to exclude the “negative control group” from the factor analysis. Nonetheless, a descriptive analysis is reported in **Supplementary Figure S2**.

### Exploratory Factor Analysis

**Figure 2:**
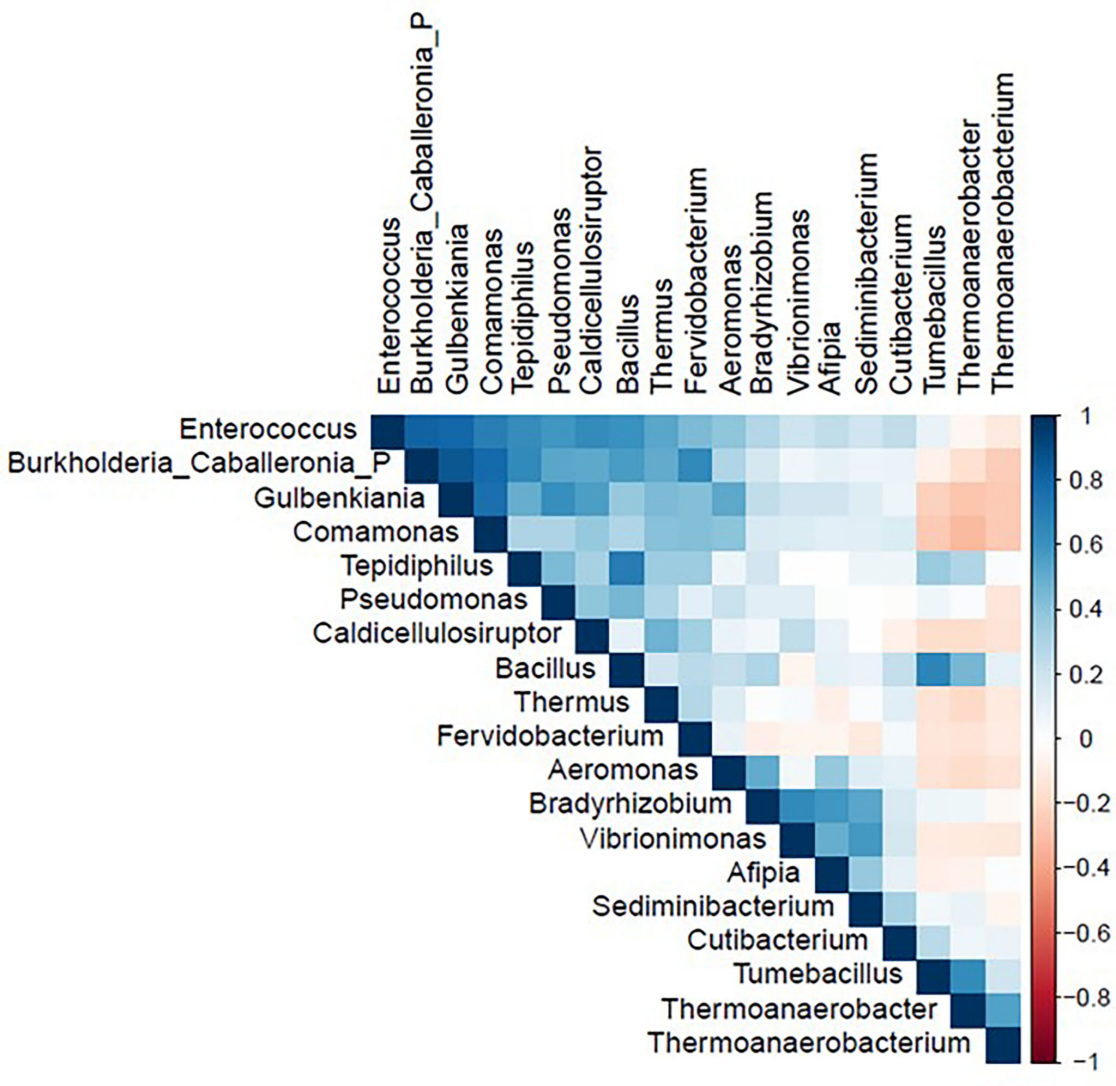
Correlation matrix of nineteen genera used in the factor analysis in the study population (N = 54). Figure generated by R software (version 4.1.2 https://www.r-project.org/).

The correlation matrix of the 19 selected genera (**Figure 2**; **Supplementary Table S5**) was suitable for factor analysis. **Table 1** reports the results of statistical procedures for checking matrix factorability. Bartlett’s test of sphericity was significant (p < 0.001). The overall measure of sampling adequacy was equal to 0.70, indicating that the sample size was sufficient, as compared to the number of genera under consideration. In addition, the individual measures of sampling adequacy were satisfactory. **Table 3** shows the factor-loading matrix for the three retained microbiome patterns, the corresponding communality estimates, and the proportion of explained variance. The retained factor explained 72.34% of the total variance in the original dataset. The first factor, named Factor1, had the highest contribution from *Enterococcus*, *Pseudomonas*, *Bacillus*, and *Burkholderia Caballeronia Paraburkholderia*. The second factor, named Factor2, was characterized by the greatest positive loadings on *Comamonas*, *Aeromonas*, *Caldicellulosiruptor*, and *Gulbenkiania* and by the highest negative loadings on *Thermoanaerobacter*, *Thermoanaerobacterium*, and *Tumebacillus*. The third pattern, named Factor3, had the highest factor loadings on *Bradyrhizobium*, *Vibrionimonas*, and *Sediminibacterium*. All the examined genera had at least one-factor loading greater than |0.40|, thus proving an important role of all genera included in this analysis.

**Table 3:** Factor-loading matrix^*^, commonalities (COMM), and explained variance for three microbiome patterns identified by factor analysis.

Genera	Factor1	Factor2	Factor3	COMM
*Aeromonas*	0.39	**0.63**	–	0.55
*Afipia*	0.16	–	0.42	0.20
*Bacillus*	**0.96**	−0.11	0.10	0.94
*Bradyrhizobium*	0.14	–	**0.91**	0.84
*Burkholderia Caballeronia Paraburkholderia*	**0.83**	0.48	0.10	0.93
*Caldicellulosiruptor*	0.35	**0.63**	–	0.54
*Comamonas*	0.34	**0.86**	–	0.85
*Cutibacterium*	0.53	–	0.22	0.33
*Enterococcus*	**0.97**	0.17	0.11	0.98
*Fervidobacterium*	0.52	0.46	–	0.48
*Gulbenkiania*	0.55	**0.66**	0.17	0.76
*Pseudomonas*	**0.74**	0.13	–	0.56
*Sediminibacterium*	–	–	**0.80**	0.66
*Tepidiphilus*	0.56	–	–	0.32
*Thermoanaerobacter*	0.22	**−0.86**	–	0.80
*Thermoanaerobacterium*	–	**−0.66**	–	0.45
*Thermus*	0.41	0.38	–	0.31
*Tumebacillus*	0.17	**−0.90**	–	0.84
*Vibrionimonas*	–	0.13	**0.99**	0.99
**Proportion of explained variance (%)**	45.23	21.40	17.06	
**Cumulative explained variance (%)**	45.23	66.63	83.69	

Loadings greater or equal to 0.63 defined dominant genera for each factor and were shown in bold typeface. Loadings smaller than |0.10| were suppressed.

^*^Estimated from a principal component factor analysis performed on 19 genera. The magnitude of each loading measures the importance of the corresponding genus to the factor.

### Effects of Nasopharyngeal Bacterial Microbiota Composition of Positive Serology Development/Maintenance

**Figure 3:**
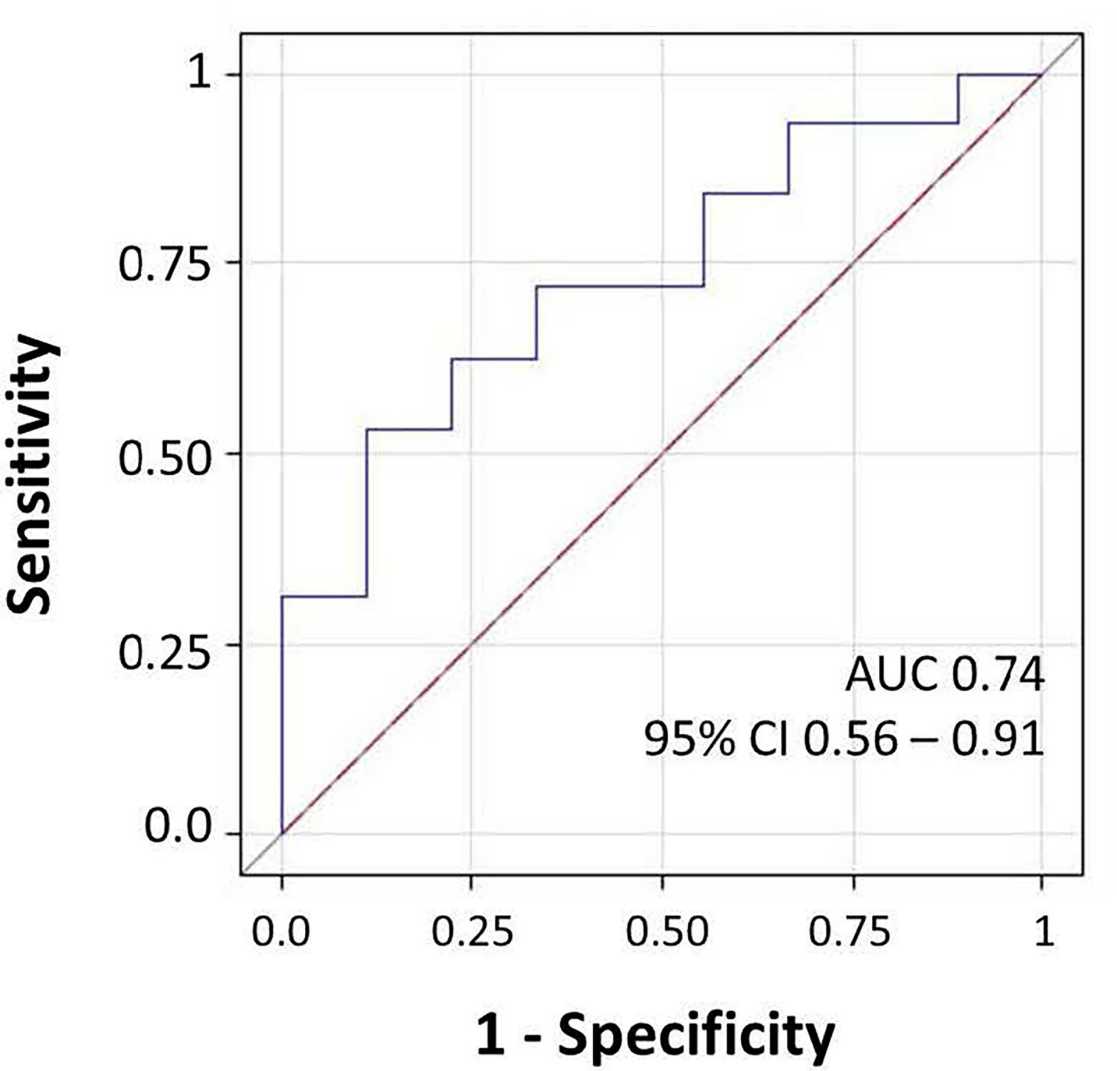
Receiver operating characteristic (ROC) curve for microbiome score for prediction of the presence of IgG at follow-up. The area under the ROC curve (AUC) and 95% CI values were annotated.

We investigated the effects of the bacterial community composition and α-diversity on the probability of developing or maintaining serum anti-RBD-IgG antibodies during the entire period of the study. No associations were observed either between the bacterial community composition or between the α-diversity indices and the probability of developing anti-RBD-IgG antibodies in the 19 participants with a positive nasal swab for SARS-CoV-2 RNA at the T1 (**Table 4** and **Supplementary Table S6**). As a sensitivity analysis, we excluded the three subjects who were negative for anti-RBD SARS-CoV-2 IgG at T1 and missing at T2. Results were comparable to those obtained in the whole group of subjects (**Supplementary Table S7**). The calculated ORs and 95% CIs of the effects of the BNM composition on maintaining a positive serology at T2 in the 41 participants with positive IgG at the T1 and with known serological anti-RBD-IgG status at the T2 are reported in **Table 5**. Shannon’s entropy α-diversity showed a positive association with serum anti-RBD-IgG antibody maintenance (OR = 5.75, 95% CI: 1.50–22.01, p = 0.0107). Factor1 pattern was positively associated with the maintenance of anti-RBD-IgG antibodies (OR = 2.64, 95% CI: 1.06–6.56, p = 0.0370). To improve the interpretability of the Factor1 pattern, we created a score by adding the relative abundance of the four Factor1 dominant genera (i.e., *Enterococcus*, *Pseudomonas*, *Bacillus*, and *Burkholderia Caballeronia Paraburkholderia*). This score was associated with a higher probability of maintaining positive IgG at the T2 (OR = 1.09, 95% CI: 1.01–1.17, p = 0.0271). Thus, the probability of maintaining anti-RBD-IgG antibodies increases by 9% for each increment of 1% in the sum of the relative abundances of the four dominant genera. When we considered single genera, only *Enterococcus* showed a positive significant association (OR = 1.21, 95% CI: 1.0–1.42, p = 0.0243) (**Supplementary Table S8**). A ROC curve was fitted to examine the prognostic ability of this score in assessing the probability to maintain anti-RBD-IgG at the T2 (**Figure 3**). The optimal threshold score was 23.3% (p = 0.0084), which yielded maximum discrimination between individuals maintaining or not the positive IgG (sensitivity 0.63, specificity 0.78).

**Table 4:** Odds ratios for the estimated contribution of each α-diversity index and microbiome pattern to the probability of developing IgG in the entire period of the study.

		OR	95% CI		p-Value	R^2^
**α-Diversity indices**	Faith pd	0.65	0.10	4.03	0.6413	0.26
	Observed features	1.02	0.89	1.16	0.7926	0.26
	Shannon entropy	0.78	0.24	2.54	0.6780	0.26
**Microbiome pattern**	Factor1	0.69	0.16	2.92	0.6168	0.26
	Factor2	0.05	0.001	9.55	0.2633	0.32
	Factor3	0.85	0.21	3.53	0.8276	0.26

The analysis was performed on 19 participants with positive SARS-CoV-2 RNA at the T1, by a multivariable logistic model adjusted for age, gender, smoking habit, and lifestyle.

**Table 5:** Odds ratios for the estimated contribution of each α-diversity index and microbiome pattern to the probability of preserving IgG antibodies at follow-up.

		OR	95% CI		p-Value	R^2^
α-Diversity indices	Faith pd	2.28	0.46	11.24	0.3113	0.18
	Observed features	1.09	0.97	1.22	0.1565	0.21
	Shannon entropy	5.75	1.50	22.01	0.0107	0.43
Microbiome pattern	Factor1	2.64	1.06	6.56	0.0370	0.33
	Factor2	0.76	0.32	1.83	0.5436	0.15
	Factor3	0.58	0.23	1.43	0.2333	0.19

The analysis was performed on 41 participants with positive IgG at T1, by a multivariable logistic model adjusted for age, gender, smoking habit, lifestyle, microbiome measured in March or May/June, and SARS-CoV-2 RNA.

## Discussion

Nasal cavities represent the principal entry and infection site of SARS-CoV-2, as most of the inhaled air enters the body through the nose and the nasal epithelium expresses high levels of the ACE2, which act as the coronavirus receptor (Hou et al., 2020). Nasopharyngeal microbiota has a critical role in protecting the host from both viral and pathogenic bacterial infections, thus cooperating with the nasal immune response (Salzano et al., 2018). In particular, the nasopharyngeal microbiota influences mucosal homeostasis (Di Stadio et al., 2020) and is involved in the development of the mucosa-associated lymphoid tissue and in the modulation of adaptive responses such as the activation of both cell-mediated and humoral immune responses (Brown et al., 2013; De Rudder et al., 2020; Dimitri-Pinheiro et al., 2020).

We characterized the BNM composition in a group of asymptomatic/paucisymptomatic individuals who tested positive for nasopharyngeal swab SARS-CoV-2 RNA and/or serum anti-RBD SARS-CoV-2 IgG at the enrolment. In terms of taxa, the BNM composition was similar to the one reported for healthy (not infected) populations of adult subjects (Man et al., 2017; Bomar et al., 2018; Mariani et al., 2018; Budden et al., 2019). Our results are supported by other previous studies reporting that patients with mild or asymptomatic COVID-19 were characterized by a bNM similar to that of negative healthy controls, suggesting that in asymptomatic/paucisymptomatic subjects who tested positive for SARS-CoV-2 RNA, the BNM composition apparently is not affected by the viral infection (De Maio et al., 2020; Rosas-Salazar et al., 2021; Shilts et al., 2022). The link between BNM composition and SARS-CoV-2 RNA has been investigated by a growing number of case–control studies that specifically focused on SARS-CoV-2-positive patients, either symptomatic or paucisymptomatic, compared to not infected healthy controls. De Maio and colleagues investigated the BNM by 16S rDNA sequencing in a group of 40 patients with mild COVID-19 disease, and no differences were observed in terms of neither the bacterial composition nor α-diversity between those who tested positive compared to those who were tested negative (De Maio et al., 2020). On the contrary, Nardelli et al. reported a significant reduction of Proteobacteria and Fusobacteria relative abundances in symptomatic patients, compared to healthy controls (Nardelli et al., 2021). The study conducted by Rueca and colleagues reported that Shannon’s α-diversity index was reduced only in patients with a severe condition requiring intensive care compared to controls and paucisymptomatic patients, thus partially supporting our results with paucisymptomatic subjects, similar to healthy controls (Rueca et al., 2021). In a recent study conducted on 103 adult subjects, ranging from asymptomatic not infective healthy subjects to very severe SARS-CoV-2-positive patients, BNM composition changes were associated with the severity of the disease, and in particular, *Corynebacterium* consistently decreased as COVID-19 severity increased (Shilts et al., 2022). In a metagenomic analysis conducted on 50 patients under investigation for COVID-19 disease, Mostafa and colleagues did not observe any significant differences at the genus and family levels but identified an α-diversity decrease in COVID-19-confirmed symptomatic patients (Mostafa et al., 2020). The partial inconsistency of these results might be due to different limitations, such as the limited number of studies in the field together with the small samples included in the analyses. Moreover, some confounders might not have been considered, such as the different pharmacological treatments and the possibility that those who were selected as negative healthy controls might have actually encountered the virus before the enrolment.

We also investigated whether BNM composition was associated with the development and/or the maintenance of serum anti-RBD-IgG antibodies. The observed positive association between α-diversity and anti-RBD-IgG antibody maintenance at the T2 suggests that the more diverse the microbiota composition, the more effective the cross-talk with the local immune component, favoring the activation of the systemic adaptive response. Indeed, lower α-diversity and richness were reported in patients with COVID-19 compared to subjects who tested negative for SARS-CoV-2 RNA in the study of Moustafa and colleagues (Mostafa et al., 2020). Since this field of research is still in its infancy, functional studies are needed to clarify the mechanisms underlying our observations.

We further applied factor analysis to group all the microbiome data information into a smaller number of independent factors able to predict the microbiome composition at the genus level by considering the relative abundances. The factorial analysis allowed us to identify three different signatures of the BNM. In particular, Factor1 was mainly characterized by *Bacillus*, *Burkholderia*, *Enterococcus*, and *Pseudomonas*, which include several opportunistic strains that may turn pathogenic and cause infections (Kumpitsch et al., 2019). Factor2 was mainly characterized by both opportunistic (such as *Aeromonas*) and environmental microbiota genera (such as *Caldicellulosibacterium* and *Comamonas*). Factor3 included different genera representative of environmental microbiota (Adams et al., 2015; Lai et al., 2017; Duan et al., 2019). In particular, this factor had the highest loading also on *Vibrionimonas*, which was the only genus that was found to be different between SARS-CoV-2 RNA-positive and RNA-negative subjects after univariate analysis. However, Factor3 was not associated either with the development or the maintenance of RBD-IgG antibodies.

Following factor analysis, we observed that the higher relative abundance of the Factor1 dominant genera was positively associated with anti-RBD-IgG maintenance. This evidence suggests that Factor1 components might influence the activation of the immune response, thus promoting the adaptive immunity against new unknown pathogens, such as the SARS-CoV-2 virus. Indeed, several species belonging to the genus *Bacillus*, such as *Bacillus subtilis*, are known stimulators of the immune system, and their colonization promotes the increase of immune cell number in the nasal mucosa, stimulating the activation of the immune response (Yang et al., 2018; Li et al., 2019). According to this hypothesis, the nasal microbiota composition was reported to influence the local host immune response and the severity of symptoms after respiratory syncytial virus bronchiolitis infection (Lynch et al., 2017; Sonawane et al., 2019; Mansbach et al., 2020; Schippa et al., 2020). Indeed, nasopharyngeal-associated lymphoid tissue (NALT), which directly interacts with the nasopharyngeal microbiota community, is constituted by a large variety and number of immune cells, including dendritic cells, macrophages, and lymphocytes (Pabst, 2015). Moreover, the BNM composition was demonstrated to influence the efficacy of a live attenuated influenza vaccine, impacting the host’s adaptive immune response and thus modulating the vaccine’s therapeutic efficacy (Salk et al., 2016). Thus, occurring shifts in the composition of the nasal microbiota may result in pro- or anti-inflammatory patterns with effects not only on the susceptibility and on the course of infection but also on the modulation of the local and systemic immune response.

We acknowledge some limitations of the present study. First, the small number of samples and the presence of potential confounders that we did not consider may have hindered the identification of distinct signatures between the different subgroups. Second, we did not assess anti-SARS-CoV-2 IgA antibodies, which play an important role in the local mucosal immunity. However, our study aimed to investigate whether the BNM composition might influence long-term immunization, which is related to IgG antibodies. Third, BNM was assessed during or after the infection; thus, we cannot exclude that we are observing the effects of the infection rather than a causal mechanism of antibody maintenance. Moreover, current guidelines are recommending to include in the airway microbiome investigations some negative controls as the gold standard. In particular, the negative sample results meaning negative from the sampling methods, the extraction process, and the PCR step should be included. In the present paper, we included negative controls to exclude any contaminations resulting from the extraction and the PCR amplification. A limitation of the study is that we did not include any sampling control. However, the main results of the paper describe an effect of Factor1, which includes strains that are not usually considered of environmental origin. Moreover, due to the pandemic context, each sampling was performed in a very controlled environment, to avoid also the SARS-CoV-2 cross-contamination of subjects (e.g., environmental disinfection after each sampling, and FFP3 masks worn by the operator and by the subjects until sampling).

## Conclusion

In conclusion, BNM is associated with the maintenance of specific anti-RBD IgG antibodies in asymptomatic/paucisymptomatic subjects, suggesting that its composition may be linked to the prompt immune activation, consequently supporting the development of immunological memory against new pathogens. To the best of our knowledge, the present study is the first to investigate the influence of BNM composition on specific IgG antibody maintenance. Further studies are required to confirm the impact of other viral infections and to unveil the mechanisms underlying the cross-talk between the BNM and the adaptive immune response.

## Data Availability Statement

The original contributions presented in the study are publicly available. This data can be found here: SRA sequenceread archive database, Accession PRJNA839581.

## Ethics Statement

The studies involving human participants were reviewed and approved by the Ethics committee of the University of Milan, Italy (approval number 17/20; approval date March 6, 2020; amendment date November 17, 2020). The patients/participants provided their written informed consent to participate in this study.

## Author Contributions

LF: study design, literature search, laboratory analysis, data interpretation, and writing. CF: data collection, statistical analysis, and writing. GS: laboratory analysis and microbiome data analysis. JM: DNA extraction and microbiome analysis. AL: microbiome analysis. MF: statistical analysis and supervision. EM: data collection, data analysis, and data interpretation. GM: study design, data collection, and funding. VB: study design, data collection, data analysis, data interpretation, and funding. UNICORN Consortium: subject’s enrolment, laboratory analysis, and data interpretation. All authors reviewed the manuscript.

## Funding

VB and GM received a grant from “Ricerche Emergenza coronavirus”, University of Milan, 2020, to support the study (https://lastatalenews.unimi.it/statale-individuati-sette-progetti-ricerca-ad-alta-priorita-contro-covid-19). Funds have been used for purchasing reagents.

## Conflict of Interest

The authors declare that the research was conducted in the absence of any commercial or financial relationships that could be construed as a potential conflict of interest.

## Publisher’s Note

All claims expressed in this article are solely those of the authors and do not necessarily represent those of their affiliated organizations, or those of the publisher, the editors and the reviewers. Any product that may be evaluated in this article, or claim that may be made by its manufacturer, is not guaranteed or endorsed by the publisher.

## Supplementary Material

Supplementary Figure 1 schematic representation of statistical analysis.

Supplementary Figure 2 Descriptive analysis of the "negative control group". **(A)** Relative abundance of the most represented genera; **(B)** alpha-diversity scores of the anti-RBD IgG positive group (IgG-pos), healthy control group (negative controls), and SARS-CoV-2 RNA positive (RNA-pos).

Supplementary Table 1 SARS-CoV-2 RNA positivity and an-ti-RBD-IgG development of the enrolled subjects during the time study title.

Supplementary Table 2 phyla relative abundance.

Supplementary Table 3 genera relative abundance.

Supplementary Table 4 Descriptive statistics of the microbiome in participants.

Supplementary Table 5 Evaluation of nineteen genera used in the factor analysis in the study population.

Supplementary Table 6 Odds ratios for the estimated contribution of each taxon at phylum and genus level to the probability of developing IgG in the entire period of the study in the 19 participants with a positive nasal swab for SARS-CoV-2 RNA at the T1.

Supplementary Table 7 Odds ratios for the estimated contribution of each taxon at phylum and genus level to the probability of developing IgG in the entire period of the study in 16 participants (we excluded the three subjects who were negative for anti-RBD SARS-CoV-2 IgG at T1 and missing at T2).

Supplementary Table 8 Odds ratios for the estimated contribution of each taxa at phylum and genus level to the probability of preserve IgG antibodies at follow-up. The analysis was performed on 41 participants with positive IgG at baseline, by multivariable logistic model adjusted for age, gender, smoking habit, lifestyle, microbiome measured in March or May/June and SARS-Cov-2 RNA. Estimates were reported for one percent increment in the relative abundance of each taxa.

## References

[B1] Adams R. I. Bateman A. C. Bik H. M. Meadow J. F. (2015). Microbiota of the Indoor Environment: A Meta-Analysis. Microbiome 3, 49. doi: 10.1186/s40168-015-0108-3 26459172 PMC4604073

[B2] Amir A. McDonald D. Navas-Molina J. A. Kopylova E. Morton J. T. Zech Xu Z.. (2017). Deblur Rapidly Resolves Single-Nucleotide Community Sequence Patterns. mSystems. 2, 1–7. doi: 10.1128/msystems.00191-16 PMC5340863 28289731

[B3] Berlin D. A. Gulick R. M. Martinez F. J. (2020). Severe Covid-19. N. Engl. J. Med. 383, 2451–2460. doi: 10.1056/nejmcp2009575 32412710

[B4] Bolyen E. Rideout J. R. Dillon M. R. Bokulich N. A. Abnet C. C. Al-Ghalith G. A.. (2019). Reproducible, Interactive, Scalable and Extensible Microbiome Data Science Using QIIME 2. Nat. Biotechnol. 37, 852–857. doi: 10.1038/s41587-019-0209-9 31341288 PMC7015180

[B5] Bomar L. Brugger S. D. Lemon K. P. (2018). Bacterial Microbiota of the Nasal Passages Across the Span of Human Life. Curr. Opin. Microbiol 41, 8–14. doi: 10.1016/j.mib.2017.10.023 29156371 PMC5862745

[B6] Brown A. F. Leech J. M. Rogers T. R. McLoughlin R. M. (2013). Staphylococcus Aureus Colonization: Modulation of Host Immune Response and Impact on Human Vaccine Design. Front. Immunol. 4, 507. doi: 10.3389/fimmu.2013.00507 PMC3884195 24409186

[B7] Budden K. F. Shukla S. D. Rehman S. F. Bowerman K. L. Keely S. Hugenholtz P.. (2019). Functional Effects of the Microbiota in Chronic Respiratory Disease. Lancet Respir. Med. 7, 907–920. doi: 10.1016/S2213-2600(18)30510-1 30975495

[B8] De Maio F. Posteraro B. Ponziani F. R. Cattani P. Gasbarrini A. Sanguinetti M. (2020). Nasopharyngeal Microbiota Profiling of SARS-CoV-2 Infected Patients. Biol. Proced. Online. 22, 1–4. doi: 10.1186/s12575-020-00131-7 32728349 PMC7382556

[B9] De Rudder C. Garcia-Tímermans C. De Boeck I. Lebeer S. Van de Wiele T. Calatayud Arroyo M. (2020). Lacticaseibacillus Casei AMBR2 Modulates the Epithelial Barrier Function and Immune Response in a Donor-Derived Nasal Microbiota Manner. Sci. Rep. 10, 1–16. doi: 10.1038/s41598-020-73857-9 33037304 PMC7547715

[B10] de Steenhuijsen Piters W. A. A. Sanders E. A. M. Bogaert D. (2015). The Role of the Local Microbial Ecosystem in Respiratory Health and Disease. Philos. Trans. R. Soc B Biol. Sci. 194, 1104–1115. doi: 10.1098/rstb.2014.0294 PMC4528492 26150660

[B11] Dimitri-Pinheiro S. Soares R. Barata P. (2020). The Microbiome of the Nose—Friend or Foe? Allergy Rhinol. 11, 1–10. doi: 10.1177/2152656720911605 PMC7074508 32206384

[B12] Di Stadio A. Costantini C. Renga G. Pariano M. Ricci G. Romani L. (2020). The Microbiota/Host Immune System Interaction in the Nose to Protect From COVID-19. Life 10, 345. doi: 10.3390/life10120345 PMC7763594 33322584

[B13] Duan S. Zhou X. Xiao H. Miao J. Zhao L. (2019). Characterization of Bacterial Microbiota in Tilapia Fillets Under Different Storage Temperatures. J. Food Sci. 84, 1487–1493. doi: 10.1111/1750-3841.14630 31066925

[B14] Gandhi R. T. Lynch J. B. del Rio C. (2020). Mild or Moderate Covid-19. N. Engl. J. Med. 383, 1757–1766. doi: 10.1056/NEJMcp2009249 32329974

[B15] Gudgeon A. C. Comrey A. L. Lee H. B. (1994). A First Course in Factor Analysis. Stat. 43, 332. doi: 10.2307/2348352

[B16] Härdle W. K. Simar L. (2012). Applied Multivariate Statistical Analysis (Berlin, Heidelberg: Springer Berlin Heidelberg). doi: 10.1007/978-3-642-17229-8

[B17] Hoffmann M. Kleine-Weber H. Schroeder S. Krüger N. Herrler T. Erichsen S.. (2020). SARS-CoV-2 Cell Entry Depends on ACE2 and TMPRSS2 and Is Blocked by a Clinically Proven Protease Inhibitor. Cell. 181, 271–280.e8. doi: 10.1016/j.cell.2020.02.052 32142651 PMC7102627

[B18] Hou Y. J. Okuda K. Edwards C. E. Martinez D. R. Asakura T. Dinnon K. H.. (2020). SARS-CoV-2 Reverse Genetics Reveals a Variable Infection Gradient in the Respiratory Tract. Cell. 182, 429–446.e14. doi: 10.1016/j.cell.2020.05.042 32526206 PMC7250779

[B19] Kumpitsch C. Koskinen K. Schöpf V. Moissl-Eichinger C. (2019). The Microbiome of the Upper Respiratory Tract in Health and Disease. BMC Biol. 17, 87. doi: 10.1186/s12915-019-0703-z 31699101 PMC6836414

[B20] Lai P. S. Allen J. G. Hutchinson D. S. Ajami N. J. Petrosino J. F. Winters T.. (2017). Impact of Environmental Microbiota on Human Microbiota of Workers in Academic Mouse Research Facilities: An Observational Study. PloS One. 12, 1–16. doi: 10.1371/journal.pone.0180969 PMC5509249 28704437

[B21] Lauer S. A. Grantz K. H. Bi Q. Jones F. K. Zheng Q. Meredith H. R.. (2020). The Incubation Period of Coronavirus Disease 2019 (CoVID-19) From Publicly Reported Confirmed Cases: Estimation and Application. Ann. Intern. Med. 172, 577–582. doi: 10.7326/M20-0504 32150748 PMC7081172

[B22] Li N. Ma W. T. Pang M. Fan Q. L. Hua J. L. (2019). The Commensal Microbiota and Viral Infection: A Comprehensive Review. Front. Immunol. 10, 1551. doi: 10.3389/fimmu.2019.01551 31333675 PMC6620863

[B23] Lynch J. P. Sikder M. A. A. Curren B. F. Werder R. B. Simpson J. Cuív P.Ó.. (2017). The Influence of the Microbiome on Early-Life Severe Viral Lower Respiratory Infections and Asthma—Food for Thought? Front. Immunol. 8. doi: 10.3389/fimmu.2017.00156 PMC5311067 28261214

[B24] Man W. H. de Steenhuijsen Piters W. A. A. Bogaert D. (2017). The Microbiota of the Respiratory Tract: Gatekeeper to Respiratory Health. Nat. Rev. Microbiol. 15, 259–270. doi: 10.1038/nrmicro.2017.14 28316330 PMC7097736

[B25] Mansbach J. M. Luna P. N. Shaw C. A. Hasegawa K. Petrosino J. F. Piedra P. A.. (2020). Increased Moraxella and Streptococcus Species Abundance After Severe Bronchiolitis is Associated With Recurrent Wheezing. J. Allergy Clin. Immunol. 145, 518–527.e8. doi: 10.1016/j.jaci.2019.10.034 31738994 PMC7010548

[B26] Man W. H. van Houten M. A. Mérelle M. E. Vlieger A. M. Chu M. L. J. N. Jansen N. J. G.. (2019). Bacterial and Viral Respiratory Tract Microbiota and Host Characteristics in Children With Lower Respiratory Tract Infections: A Matched Case-Control Study. Lancet Respir. Med. 7, 417–426. doi: 10.1016/S2213-2600(18)30449-1 30885620 PMC7172745

[B27] Mariani J. Favero C. Spinazzè A. Cavallo D. M. Carugno M. Motta V.. (2018). Short-Term Particulate Matter Exposure Influences Nasal Microbiota in a Population of Healthy Subjects. Environ. Res. 162, 119–126. doi: 10.1016/j.envres.2017.12.016 29291434

[B28] Mazzini L. Martinuzzi D. Hyseni I. Benincasa L. Molesti E. Casa E.. (2021). Comparative Analyses of SARS-CoV-2 Binding (IgG, IgM, IgA) and Neutralizing Antibodies From Human Serum Samples. Nat Libr Med. 489, 112937. doi: 10.1016/j.jim.2020.112937 PMC7695554 33253698

[B29] Milani G. P. Dioni L. Favero C. Cantone L. Macchi C. Delbue S.. (2020a). Serological Follow-Up of SARS-CoV-2 Asymptomatic Subjects. Sci. Rep. 10, 1–7. doi: 10.1038/s41598-020-77125-8 33208819 PMC7674414

[B30] Milani G. P. Montomoli E. Bollati V. Albetti B. Bandi C. Bellini T.. (2020b). SARS-CoV-2 Infection Among Asymptomatic Homebound Subjects in Milan, Italy. Eur. J. Intern. Med. 78, 161–163. doi: 10.1016/j.ejim.2020.06.010 32564906 PMC7280100

[B31] Milani G. P. Rota F. Favero C. Dioni L. Manenti A. Hoxha M.. (2021). Detection of IgM, IgG and SARS-CoV-2 RNA Among the Personnel of the University of Milan, March Through May 2020: The UNICORN Study. BMJ Open 11:1–8. doi: 10.1136/BMJOPEN-2020-046800 PMC7992385 33762247

[B32] Mostafa H. H. Fissel J. A. Fanelli B. Bergman Y. Gniazdowski V. Dadlani M.. (2020). Metagenomic Next-Generation Sequencing of Nasopharyngeal Specimens Collected From Confirmed and Suspect Covid-19 Patients. MBio. 11, 1–13. doi: 10.1128/mBio.01969-20 PMC7686804 33219095

[B33] Nardelli C. Gentile I. Setaro M. Di Domenico C. Pinchera B. Buonomo A. R.. (2021). Nasopharyngeal Microbiome Signature in COVID-19 Positive Patients: Can We Definitively Get a Role to Fusobacterium Periodonticum? Front. Cell. Infect. Microbiol. 11. doi: 10.3389/fcimb.2021.625581 PMC7919745 33659220

[B34] Pabst R. (2015). Mucosal Vaccination by the Intranasal Route. Nose-Associated Lymphoid Tissue (NALT)-Structure, Function and Species Differences. Vaccine. 33, 4406–4413. doi: 10.1016/j.vaccine.2015.07.022 26196324

[B35] Rajalahti T. Kvalheim O. M. (2011). Multivariate Data Analysis in Pharmaceutics: A Tutorial Review. Int. J. Pharm. 417, 280–290. doi: 10.1016/j.ijpharm.2011.02.019 21335075

[B36] Rodda L. B. Netland J. Shehata L. Pruner K. B. Morawski P. A. Thouvenel C. D.. (2021). Functional SARS-CoV-2-Specific Immune Memory Persists After Mild COVID-19. Cell. 184, 169–183.e17. doi: 10.1016/j.cell.2020.11.029 33296701 PMC7682481

[B37] Rognes T. Flouri T. Nichols B. Quince C. Mahé F. (2016). VSEARCH: A Versatile Open Source Tool for Metagenomics. PeerJ 4, e2584. doi: 10.7717/peerj.2584 27781170 PMC5075697

[B38] Rosas-Salazar C. Kimura K. S. Shilts M. H. Strickland B. A. Freeman M. H. Wessinger B. C.. (2021). SARS-CoV-2 Infection and Viral Load are Associated With the Upper Respiratory Tract Microbiome. J. Allergy Clin. Immunol. 147, 1226–1233.e2. doi: 10.1016/J.JACI.2021.02.001 33577896 PMC7871823

[B39] Rueca M. Fontana A. Bartolini B. Piselli P. Mazzarelli A. Copetti M.. (2021). Investigation of Nasal/Oropharyngeal Microbial Community of COVID-19 Patients by 16S rDNA Sequencing. Int. J. Environ. Res. Public Health 18, 1–12. doi: 10.3390/IJERPH18042174 PMC7926517 33672177

[B40] Salk H. M. Simon W. L. Lambert N. Kennedy R. B. Grill D. E. Kabat B. F.. (2016). Taxa of the Nasal Microbiome are Associated With Influenza-Specificiga Response to Live Attenuated Influenza Vaccine. PloS One. 11, 1–13. doi: 10.1371/journal.pone.0162803 PMC5028048 27643883

[B41] Salzano F. A. Marino L. Salzano G. Botta R. M. Cascone G. D’Agostino Fiorenza U.. (2018). Microbiota Composition and the Integration of Exogenous and Endogenous Signals in Reactive Nasal Inflammation. J. Immunol. Res. 2018, 1–17. doi: 10.1155/2018/2724951 PMC6008798 29967798

[B42] Schippa S. Frassanito A. Marazzato M. Nenna R. Petrarca L. Neroni B.. (2020). Nasal Microbiota in RSV Bronchiolitis. Microorganisms 8, 731. doi: 10.3390/microorganisms8050731 PMC7284514 32414211

[B43] Shilts M. H. Rosas-Salazar C. Strickland B. A. Kimura K. S. Asad M. Sehanobish E.. (2022). Severe COVID-19 Is Associated With an Altered Upper Respiratory Tract Microbiome. Front. Cell. Infect. Microbiol. 11. doi: 10.3389/FCIMB.2021.781968 PMC8819187 35141167

[B44] Sonawane A. R. Tian L. Chu C. Y. Qiu X. Wang L. Holden-Wiltse J.. (2019). Microbiome-Transcriptome Interactions Related to Severity of Respiratory Syncytial Virus Infection. Sci. Rep. 9, 1–14. doi: 10.1038/s41598-019-50217-w 31554845 PMC6761288

[B45] Sun K. Chen J. Viboud C. (2020). Early Epidemiological Analysis of the Coronavirus Disease 2019 Outbreak Based on Crowdsourced Data: A Population-Level Observational Study. Lancet Digit. Heal. 2 e201–e208. doi: 10.1016/S2589-7500(20)30026-1 PMC7158945 32309796

[B46] Tay M. Z. Poh C. M. Rénia L. MacAry P. A. Ng L. F. P. (2020). The Trinity of COVID-19: Immunity, Inflammation and Intervention. Nat. Rev. Immunol. 20, 363–374. doi: 10.1038/s41577-020-0311-8 32346093 PMC7187672

[B47] Vicenzi M. Di Cosola R. Ruscica M. Ratti A. Rota I. Rota F.. (2020). The Liaison Between Respiratory Failure and High Blood Pressure: Evidence From COVID-19 Patients. Eur. Respir. J. 51, 1–4. doi: 10.1183/13993003.01157-2020 PMC7241109 32430432

[B48] WHO (2021). COVID-19 Weekly Epidemiological Update. World Heal. Organ., 1–23. Available at: https://www.who.int/emergencies/diseases/novel-coronavirus-2019/situation-reports

[B49] Wu Z. McGoogan J. M. (2020). Characteristics of and Important Lessons From the Coronavirus Disease 2019 (COVID-19) Outbreak in China. JAMA 323, 1239. doi: 10.1001/jama.2020.2648 32091533

[B50] Yang Y. Jing Y. Yang J. Yang Q. (2018). Effects of Intranasal Administration With Bacillusï¿½subtilis on Immune Cells in the Nasal Mucosa and Tonsils of Piglets. Exp. Ther. Med. 159, 156–166. doi: 10.3892/etm.2018.6093 PMC5958783 29805543

