# Supplementary figures and images for: Nasopharyngeal Bacterial Microbiota Composition and SARS-CoV-2 IgG Antibody Maintenance in Asymptomatic/Paucisymptomatic Subjects
Source: Front Cell Infect Microbiol. 2022 Jul 6;12:882302. doi: 10.3389/fcimb.2022.882302 (PMC9297915; doi:10.3389/fcimb.2022.882302)

***Supplementary Figure S1:*** *schematic representation of statistical analysis.*


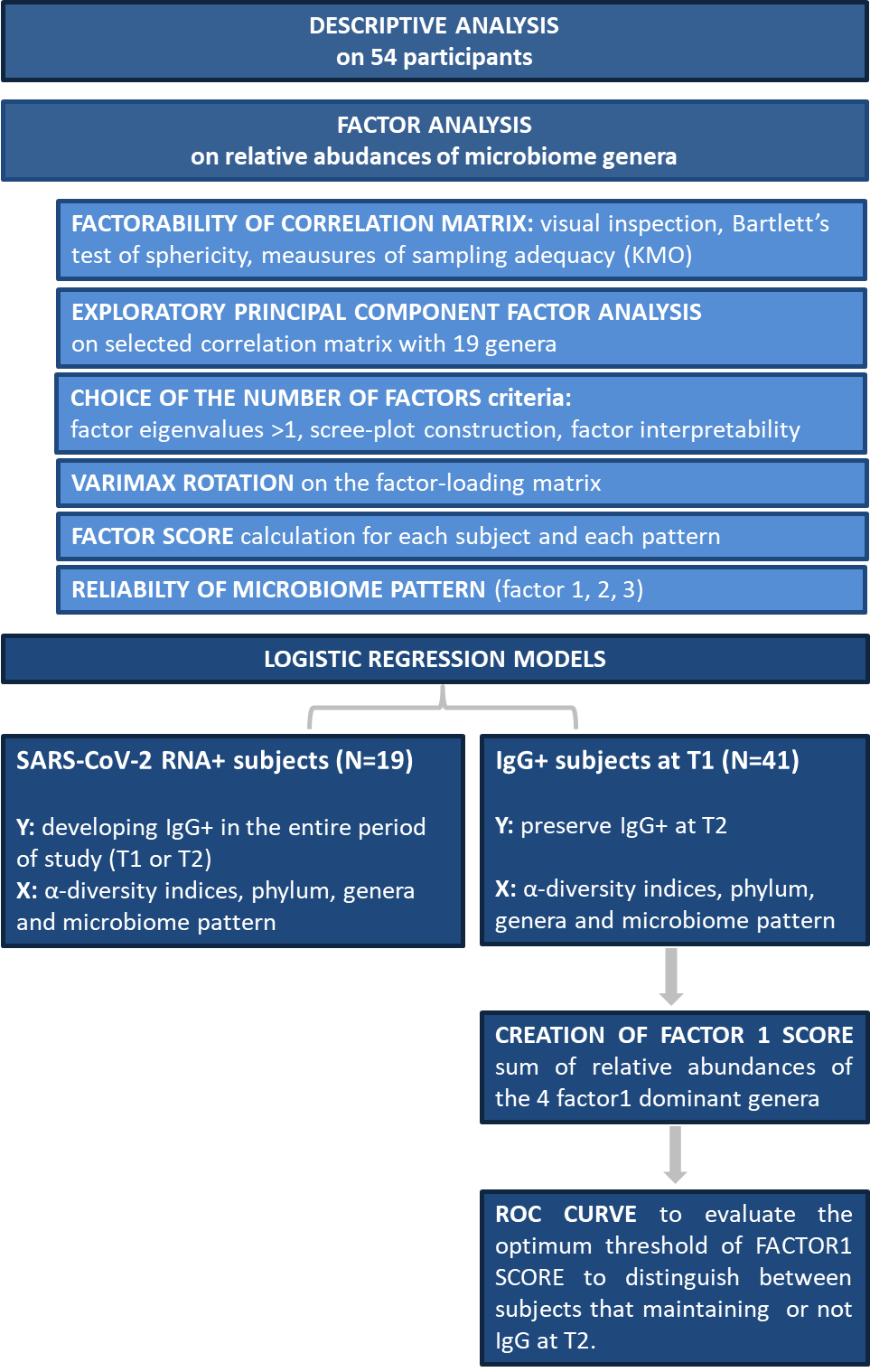

Supplement: Supplementary Figure 1 — schematic representation of statistical analysis. [file DataSheet_1.docx]

a

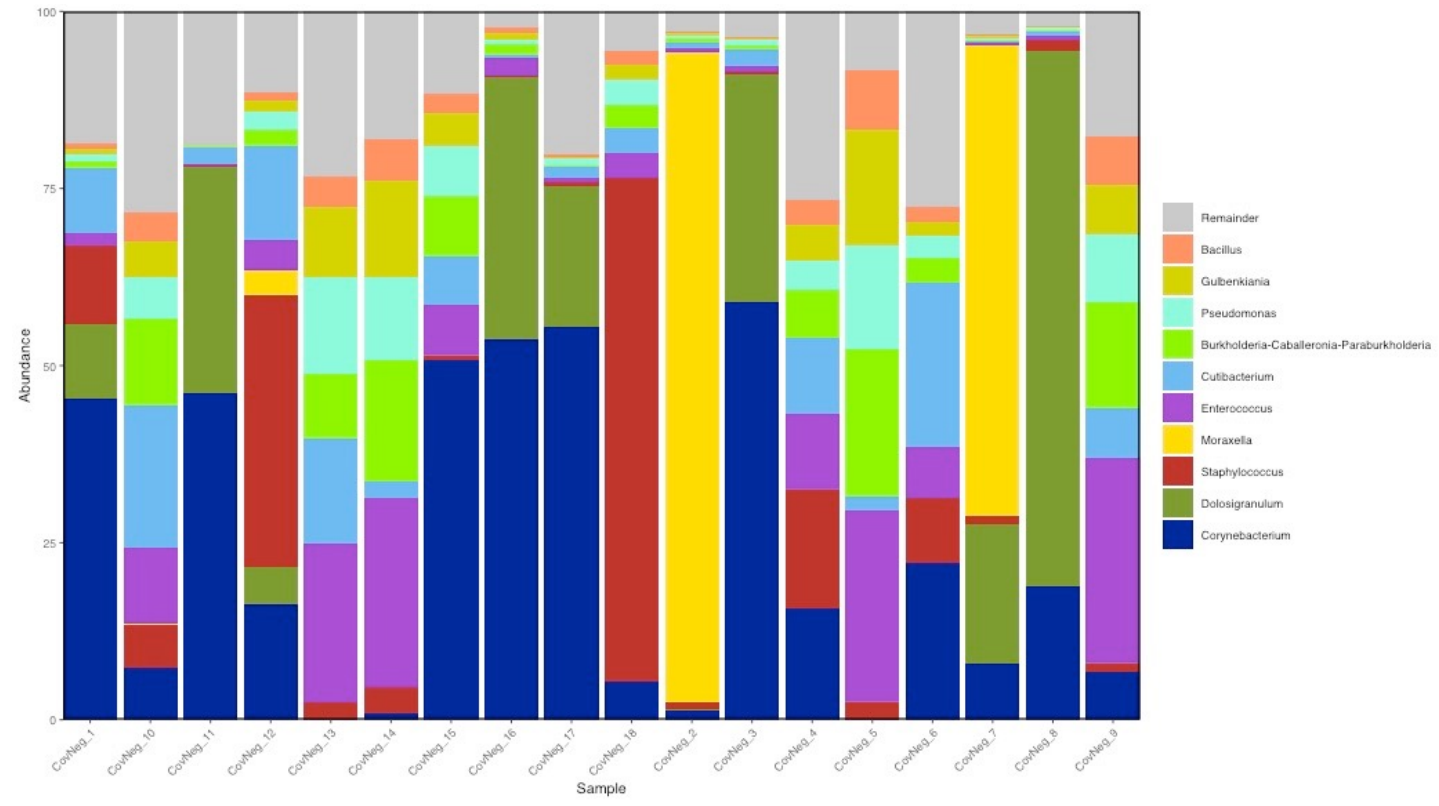

b

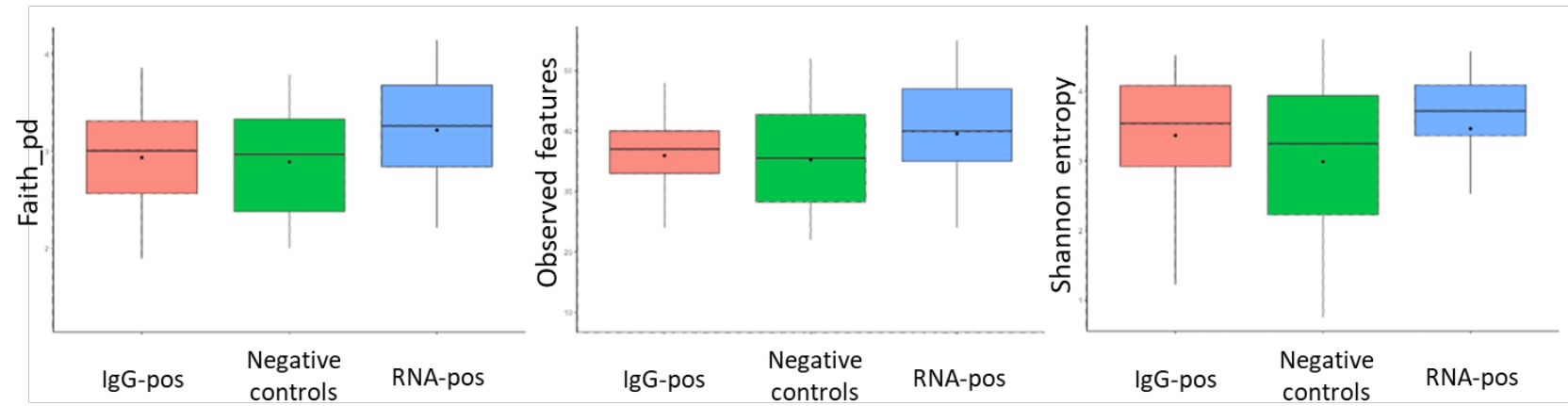

Supplement: Supplementary Figure 2 — Descriptive analysis of the "negative control group". (A) Relative abundance of the most represented genera; (B) alpha-diversity scores of the anti-RBD IgG positive group (IgG-pos), healthy control group (negative controls), and SARS-CoV-2 RNA positive (RNA-pos). [file DataSheet_2.pdf]
