# Supplementary material for: Nasopharyngeal Bacterial Microbiota Composition and SARS-CoV-2 IgG Antibody Maintenance in Asymptomatic/Paucisymptomatic Subjects
Source: Front Cell Infect Microbiol. 2022 Jul 6;12:882302. doi: 10.3389/fcimb.2022.882302 (PMC9297915; doi:10.3389/fcimb.2022.882302)
Supplement: Supplementary Table 1 — SARS-CoV-2 RNA positivity and an-ti-RBD-IgG development of the enrolled subjects during the time study title. [file Table_1.docx]

**Supplementary Table S1:** SARS-CoV-2 RNA positivity and anti-RBD-IgG development of the enrolled subjects during the time study.

| **Sample ID** | **SARS-CoV-2 RNA+** | **anti-RBD-IgG+ at the T1** | **anti-RBD-IgG+ at the T2** | **ever developed anti-RBD-IgG+** |
| --- | --- | --- | --- | --- |
| 1 | + | - | - | - |
| 2 | + | + | + | + |
| 3 | + | + | n.a. | + |
| 4 | + | - | - | - |
| 5 | + | + | + | + |
| 6 | + | - | n.a. | - |
| 7 | + | + | + | + |
| 8 | + | - | - | - |
| 9 | + | - | n.a. | - |
| 10 | + | - | + | + |
| 11 | + | - | - | - |
| 12 | + | - | - | - |
| 13 | + | - | - | - |
| 14 | + | - | - | - |
| 15 | + | + | - | + |
| 16 | + | + | + | + |
| 17 | + | - | n.a. | - |
| 18 | + | + | + | + |
| 19 | + | + | n.a. | + |
| 20 | - | + | + | + |
| 21 | - | + | + | + |
| 22 | - | + | + | + |
| 23 | - | + | - | + |
| 24 | - | + | - | + |
| 25 | - | + | + | + |
| 26 | - | + | + | + |
| 27 | - | + | + | + |
| 28 | - | + | + | + |
| 29 | - | + | + | + |
| 30 | - | + | + | + |
| 31 | - | + | + | + |
| 32 | - | + | + | + |
| 33 | - | + | + | + |
| 34 | - | + | + | + |
| 35 | - | + | - | + |
| 36 | - | + | - | + |
| 37 | - | + | + | + |
| 38 | - | + | + | + |
| 39 | - | + | + | + |
| 40 | - | + | + | + |
| 41 | - | + | + | + |
| 42 | - | + | - | + |
| 43 | - | + | + | + |
| 44 | - | + | - | + |
| 45 | - | + | - | + |
| 46 | - | + | + | + |
| 47 | - | + | + | + |
| 48 | - | + | + | + |
| 49 | - | + | - | + |
| 50 | - | + | + | + |
| 51 | - | + | + | + |
| 52 | - | + | + | + |
| 53 | - | + | + | + |
| 54 | - | + | + | + |

+: positive; -: negative; n.a. not availableavaliable
