# Supplementary material for: Nasopharyngeal Bacterial Microbiota Composition and SARS-CoV-2 IgG Antibody Maintenance in Asymptomatic/Paucisymptomatic Subjects
Source: Front Cell Infect Microbiol. 2022 Jul 6;12:882302. doi: 10.3389/fcimb.2022.882302 (PMC9297915; doi:10.3389/fcimb.2022.882302)
Supplement: Supplementary Table 5 — Evaluation of nineteen genera used in the factor analysis in the study population. [file Table_5.docx]

**Supplementary table S5.** Evaluation of nineteen genera used in the factor analysis in the study population (N=54). Variables were not normally distributed.

| **Genera** | **Number of subjects with genera not expressed** | **Median [Q1;Q3]** |
| --- | --- | --- |
| *Aeromonas* | 19 (35.2%) | 0.25 [0.12;0.54] |
| *Afipia* | 43 (79.6%) | 0.33 [0.06;0.73] |
| *Bacillus* | 0 (0.0%) | 3.16 [1.37;6.17] |
| *Bradyrhizobium* | 17 (31.5%) | 1.01 [0.35;2.6] |
| *Burkholderia Caballeronia Parabulkholderia* | 0 (0.0%) | 3.79 [0.89;8.61] |
| *Caldicellulosiruptor* | 18 (33.3%) | 0.48 [0.11;0.71] |
| *Comamonas* | 13 (24.1%) | 0.6 [0.24;1.15] |
| *Cutibacterium* | 0 (0.0%) | 3.77 [1.45;7.98] |
| *Enterococcus* | 0 (0.0%) | 6.63 [3.41;16.53] |
| *Fervidobacterium* | 20 (37.0%) | 0.12 [0.04;0.29] |
| *Gulbenkiania* | 1 (1.9%) | 2.23 [0.78;5.43] |
| *Pseudomonas* | 0 (0.0%) | 6.97 [2.46;12.24] |
| *Sediminibacterium* | 27 (50%) | 0.37 [0.1;0.95] |
| *Tepidiphilus* | 5 (9.3%) | 0.78 [0.24;1.95] |
| *Thermoanaerobacter* | 42 (77.8%) | 0.37 [0.12;0.83] |
| *Thermoanaerobacterium* | 45 (83.3%) | 0.21 [0.07;0.73] |
| *Thermus* | 23 (42.6%) | 0.14 [0.04;0.65] |
| *Tumebacillus* | 46 (85.2%) | 0.37 [0.25;1.87] |
| *Vibrionimonas* | 20 (37.0%) | 0.7 [0.28;1.78] |

Q1: First quartile, Q3: Third quartile.
