# Supplementary material for: Nasopharyngeal Bacterial Microbiota Composition and SARS-CoV-2 IgG Antibody Maintenance in Asymptomatic/Paucisymptomatic Subjects
Source: Front Cell Infect Microbiol. 2022 Jul 6;12:882302. doi: 10.3389/fcimb.2022.882302 (PMC9297915; doi:10.3389/fcimb.2022.882302)
Supplement: Supplementary Table 6 — Odds ratios for the estimated contribution of each taxon at phylum and genus level to the probability of developing IgG in the entire period of the study in the 19 participants with a positive nasal swab for SARS-CoV-2 RNA at the T1. [file Table_6.docx]

**Supplementary Table S6.** Odds ratios for the estimated contribution of each taxa at phylumand genus level to the probability of develop IgG in the entire period of the study. The analysis was performed on 19 participants with positive SARS-Cov-2 RNA, by multivariable logistic model adjusted for age, gender, smoking habit and lifestyle. Estimates were reported for one percent increment in the relative abundance of each taxa.

|  |  | **OR** | **95% CI** | | **P-value** | **FDR  P-value** | **R^2^** |
| --- | --- | --- | --- | --- | --- | --- | --- |
| **L1 Phylum** | *Actinobacteria* | 1.04 | 0.98 | 1.11 | 0.1879 | 0.4385 | 0.45 |
|  | *Bacteroidetes* | 0.52 | 0.23 | 1.22 | 0.1330 | 0.4385 | 0.49 |
|  | *Deinococcus-Thermus* | 2.16 | 0.08 | 56.25 | 0.6424 | 0.8993 | 0.35 |
|  | *Epsilonbacteraeota* | 0.84 | 0.11 | 6.52 | 0.8679 | 0.9915 | 0.34 |
|  | *Firmicutes* | 1.00 | 0.94 | 1.07 | 0.9915 | 0.9915 | 0.34 |
|  | *Proteobacteria* | 0.95 | 0.88 | 1.02 | 0.1758 | 0.4385 | 0.46 |
|  | *Thermotogae* | 0.10 | <0.001 | 47.09 | 0.4620 | 0.8086 | 0.37 |
| **L5 Genera** | *Corynebacterium 1* | 1.03 | 0.98 | 1.09 | 0.2591 | 0.9241 | 0.41 |
|  | *Lawsonella* | 1.29 | 0.87 | 1.92 | 0.2001 | 0.9241 | 0.44 |
|  | *Micrococcus* | 0.01 | <0.001 | >999 | 0.5741 | 0.9394 | 0.36 |
|  | *Cutibacterium* | 0.99 | 0.76 | 1.29 | 0.9321 | 0.9821 | 0.34 |
|  | *Sediminibacterium* | 0.08 | 0.00 | 4.02 | 0.2089 | 0.9241 | 0.43 |
|  | *Vibrionimonas* | 0.48 | 0.18 | 1.26 | 0.1344 | 0.9241 | 0.49 |
|  | *Deinococcus* | <0.001 | <0.001 | 367.34 | 0.1924 | 0.9241 | 0.44 |
|  | *Thermus* | 5.03 | 0.10 | 252.62 | 0.4189 | 0.9241 | 0.38 |
|  | *Campylobacter* | 0.84 | 0.11 | 6.52 | 0.8679 | 0.9821 | 0.34 |
|  | *Tumebacillus* | 57.49 | <0.001 | >999 | 0.9802 | 0.9821 | 0.36 |
|  | *Bacillus* | 0.95 | 0.70 | 1.28 | 0.7352 | 0.9821 | 0.35 |
|  | *Geobacillus* | <0.001 | <0.001 | >999 | 0.6394 | 0.9821 | 0.73 |
|  | *Gemella* | 149.12 | <0.001 | >999 | 0.8512 | 0.9821 | 0.34 |
|  | *Staphylococcus* | 0.89 | 0.73 | 1.08 | 0.2384 | 0.9241 | 0.43 |
|  | *Carnobacterium* | 8.44 | 0.13 | 546.73 | 0.3161 | 0.9241 | 0.42 |
|  | *Dolosigranulum* | 1.03 | 0.96 | 1.12 | 0.3790 | 0.9241 | 0.39 |
|  | *Enterococcus* | 1.00 | 0.84 | 1.18 | 0.9807 | 0.9821 | 0.34 |
|  | *Streptococcus* | 1.06 | 0.65 | 1.72 | 0.8259 | 0.9821 | 0.34 |
|  | *Clostridium sensu stricto 10* | 0.21 | <0.001 | 326.48 | 0.6736 | 0. 9394 | 0.35 |
|  | *Anaerococcus* | 0.59 | 0.19 | 1.79 | 0.3496 | 0.9241 | 0.39 |
|  | *Finegoldia* | 0.61 | 0.27 | 1.36 | 0.2273 | 0.9241 | 0.48 |
|  | *Peptoniphilus* | 0.75 | 0.44 | 1.29 | 0.2961 | 0.9821 | 0.40 |
|  | *Caldicellulosiruptor* | 0.40 | 0.003 | 56.02 | 0.7149 | 0.9821 | 0.35 |
|  | *Thermoanaerobacterium* | >999 | <0.001 | <0.001 | 0.9801 | 0.9821 | 0.36 |
|  | *Thermoanaerobacter* | >999 | <0.001 | >999 | 0.9802 | 0.9821 | 0.36 |
|  | *Thermosinus* | >999 | <0.001 | >999 | 0.7651 | 0.9394 | 0.41 |
|  | *Labrys* | 933.64 | <0.001 | >999 | 0.5745 | 0.9394 | 0.36 |
|  | *Mesorhizobium* | 370.62 | <0.001 | >999 | 0.5267 | 0.9241 | 0.47 |
|  | *Afipia* | 0.05 | <0.001 | 61.29 | 0.4114 | 0.9241 | 0.39 |
|  | *Bradyrhizobium* | 0.71 | 0.30 | 1.68 | 0.4383 | 0.9241 | 0.37 |
|  | *Paracoccus* | 0.73 | 0.004 | 124.99 | 0.9027 | 0.9821 | 0.34 |
|  | *Sphingomonas* | <0.001 | <0.001 | <0.001 | 0.8471 | 0.9821 | 0.69 |
|  | *Aeromonas* | >999 | 0.17 | >999 | 0.1122 | 0.9241 | 0.54 |
|  | *Burkholderia-Caballeronia-Paraburkholderia* | 0.92 | 0.71 | 1.20 | 0.5246 | 0.9394 | 0.36 |
|  | *Comamonas* | 0.34 | 0.03 | 4.57 | 0.4147 | 0.9821 | 0.38 |
|  | *Ralstonia* | 1.06 | 0.01 | 208.70 | 0.9821 | 0.9394 | 0.34 |
|  | *Gulbenkiania* | 0.88 | 0.57 | 1.35 | 0.5530 | 0.9394 | 0.36 |
|  | *Tepidiphilus* | 1.55 | 0.51 | 4.74 | 0.4430 | 0.9241 | 0.38 |
|  | *Citrobacter* | 2.51 | <0.001 | >999 | 0.8501 | 0.9821 | 0.34 |
|  | *Escherichia-Shigella* | 0.10 | 0.003 | 3.42 | 0.2016 | 0.9241 | 0.46 |
|  | *Serratia* | 0.62 | 0.12 | 3.27 | 0.5722 | 0.9394 | 0.36 |
|  | *Acinetobacter* | 1.47 | 0.08 | 27.07 | 0.7940 | 0.9821 | 0.34 |
|  | *Enhydrobacter* | >999 | 0.005 | >999 | 0.1853 | 0.9241 | 0.48 |
|  | *Moraxella* | 0.97 | 0.88 | 1.08 | 0.5871 | 0.9394 | 0.37 |
|  | *Pseudomonas* | 0.88 | 0.73 | 1.08 | 0.2169 | 0.9241 | 0.43 |
|  | *Fervidobacterium* | 0.10 | <0.001 | 47.09 | 0.4620 | 0.9241 | 0.37 |
