# Supplementary material for: Nasopharyngeal Bacterial Microbiota Composition and SARS-CoV-2 IgG Antibody Maintenance in Asymptomatic/Paucisymptomatic Subjects
Source: Front Cell Infect Microbiol. 2022 Jul 6;12:882302. doi: 10.3389/fcimb.2022.882302 (PMC9297915; doi:10.3389/fcimb.2022.882302)
Supplement: Supplementary Table 7 — Odds ratios for the estimated contribution of each taxon at phylum and genus level to the probability of developing IgG in the entire period of the study in 16 participants (we excluded the three subjects who were negative for anti-RBD SARS-CoV-2 IgG at T1 and missing at T2). [file Table_7.docx]

**Supplementary Table S7:** The analysis was performed on 16 participants with positive SARS-Cov-2 RNA at the T1, by a multivariable logistic model adjusted for age, gender, smoking habit, and lifestyle.

|  |  | **OR** | **95% CI** | | **P-value** | **R^2^** |
| --- | --- | --- | --- | --- | --- | --- |
| **α- diversity indices** | faith pd | 0.043 | <0.001 | 8.17 | 0.2393 | 0.54 |
|  | observed features | 0.86 | 0.60 | 1.23 | 0.4121 | 0.50 |
|  | shannon entropy | 0.38 | 0.035 | 4.09 | 0.4233 | 0.49 |
|  |  |  |  |  |  |  |
| **Microbiome pattern** | Factor1 | 0.39 | 0.03 | 5.12 | 0.4775 | 0.48 |
|  | Factor2 | 2.61 | 0.002 | 999.9 | 0.7986 | 0.46 |
|  | Factor3 | 0.002 | <0.001 | 5.57 | 0.1272 | 0.64 |
