# Supplementary material for: Nasopharyngeal Bacterial Microbiota Composition and SARS-CoV-2 IgG Antibody Maintenance in Asymptomatic/Paucisymptomatic Subjects
Source: Front Cell Infect Microbiol. 2022 Jul 6;12:882302. doi: 10.3389/fcimb.2022.882302 (PMC9297915; doi:10.3389/fcimb.2022.882302)
Supplement: Supplementary Table 8 — Odds ratios for the estimated contribution of each taxa at phylum and genus level to the probability of preserve IgG antibodies at follow-up. The analysis was performed on 41 participants with positive IgG at baseline, by multivariable logistic model adjusted for age, gender, smoking habit, lifestyle, microbiome measured in March or May/June and SARS-Cov-2 RNA. Estimates were reported for one percent increment in the relative abundance of each taxa. [file Table_8.docx]

**Supplementary Table S8.** Odds ratios for the estimated contribution of each taxa at phylum and genus level to the probability of preserve IgG antibodies at follow-up. The analysis was performed on 41 participants with positive IgG at baseline, by multivariable logistic model adjusted for age, gender, smoking habit, lifestyle, microbiome measured in March or May/June and SARS-Cov-2 RNA. Estimates were reported for one percent increment in the relative abundance of each taxa.

|  |  | **OR** | **95% CI** | | **P-value** | **FDR P-value** | **R^2^** |
| --- | --- | --- | --- | --- | --- | --- | --- |
| **L1 Phylum** | *Actinobacteria* | 0.97 | 0.94 | 1.01 | 0.1198 | 0.4193 | 0.23 |
|  | *Bacteroidetes* | 0.91 | 0.51 | 1.61 | 0.7369 | 0.9463 | 0.15 |
|  | *Deinococcus-Thermus* | 3.34 | 0.54 | 20.50 | 0.1932 | 0.4507 | 0.22 |
|  | *Epsilonbacteraeota* | 1.27 | 0.10 | 16.76 | 0.8567 | 0.9463 | 0.14 |
|  | *Firmicutes* | 1.00 | 0.94 | 1.06 | 0.9463 | 0.9463 | 0.14 |
|  | *Proteobacteria* | 1.05 | 0.99 | 1.12 | 0.0735 | 0.4193 | 0.29 |
|  | *Thermotogae* | 0.77 | 0.07 | 8.06 | 0.8253 | 0.9463 | 0.14 |
| **L5 Genera** | *Corynebacterium 1* | 0.98 | 0.95 | 1.01 | 0.1598 | 0.6727 | 0.21 |
|  | *Lawsonella* | 0.96 | 0.77 | 1.20 | 0.7370 | 0.9125 | 0.15 |
|  | *Micrococcus* | >999 | <0.001 | >999 | 0.8789 | 0.9163 | 0.28 |
|  | *Cutibacterium* | 0.98 | 0.88 | 1.09 | 0.7357 | 0.9125 | 0.15 |
|  | *Sediminibacterium* | 0.51 | 0.06 | 4.29 | 0.5343 | 0.8909 | 0.15 |
|  | *Vibrionimonas* | 0.93 | 0.46 | 1.89 | 0.8347 | 0.9125 | 0.14 |
|  | *Deinococcus* | 2.13 | 0.18 | 24.50 | 0.5454 | 0.8909 | 0.16 |
|  | *Thermus* | 16.65 | 0.31 | 880.08 | 0.1648 | 0.6727 | 0.26 |
|  | *Campylobacter* | 1.27 | 0.10 | 16.76 | 0.8567 | 0.9125 | 0.14 |
|  | *Tumebacillus* | 3.44 | 0.12 | 99.93 | 0.4723 | 0.8571 | 0.19 |
|  | ***Bacillus*** | **1.46** | **1.00** | **2.14** | **0.0518** | **0.5589** | **0.31** |
|  | *Geobacillus* | 599.95 | <0.001 | >999 | 0.2563 | 0.7617 | 0.24 |
|  | *Gemella* | 0.51 | 0.06 | 4.35 | 0.5357 | 0.8909 | 0.15 |
|  | *Staphylococcus* | 0.96 | 0.90 | 1.02 | 0.1907 | 0.7189 | 0.20 |
|  | *Carnobacterium* | 7.68 | 0.09 | 657.89 | 0.3693 | 0.8064 | 0.18 |
|  | *Dolosigranulum* | 0.97 | 0.93 | 1.02 | 0.2734 | 0.7617 | 0.18 |
|  | ***Enterococcus*** | **1.21** | **1.02** | **1.42** | **0.0243** | **0.5589** | **0.37** |
|  | *Streptococcus* | 1.07 | 0.77 | 1.47 | 0.6998 | 0.9125 | 0.15 |
|  | *Clostridium sensu stricto 10* | >999 | 0.002 | >999 | 0.2950 | 0.7617 | 0.20 |
|  | *Anaerococcus* | 1.38 | 0.66 | 2.87 | 0.3950 | 0.8064 | 0.18 |
|  | *Finegoldia* | 3.61 | 0.39 | 33.64 | 0.2596 | 0.7617 | 0.22 |
|  | *Peptoniphilus* | 0.98 | 0.56 | 1.71 | 0.9480 | 0.948 | 0.14 |
|  | *Caldicellulosiruptor* | 9.78 | 0.67 | 143.17 | 0.0959 | 0.5589 | 0.28 |
|  | *Thermoanaerobacterium* | 0.73 | 0.04 | 13.50 | 0.8294 | 0.9125 | 0.14 |
|  | *Thermoanaerobacter* | 13.25 | 0.12 | 1438.24 | 0.2798 | 0.7617 | 0.20 |
|  | *Thermosinus* | 1.82 | 0.47 | 7.10 | 0.3885 | 0.8064 | 0.17 |
|  | *Labrys* | 0.57 | <0.01 | 187.65 | 0.8511 | 0.9125 | 0.14 |
|  | *Mesorhizobium* | 36.01 | <0.001 | >999 | 0.7641 | 0.9125 | 0.16 |
|  | *Afipia* | 0.53 | <0.01 | 244.66 | 0.8400 | 0.9125 | 0.14 |
|  | *Bradyrhizobium* | 0.98 | 0.49 | 1.93 | 0.9424 | 0.948 | 0.14 |
|  | *Paracoccus* | >999 | <0.001 | >999 | 0.2630 | 0.7617 | 0.30 |
|  | *Sphingomonas* | >999 | <0.001 | >999 | 0.6775 | 0.9125 | 0.20 |
|  | *Aeromonas* | 1.28 | 0.13 | 12.36 | 0.8283 | 0.9125 | 0.14 |
|  | ***Burkholderia-Caballeronia-Paraburkholderia*** | **1.22** | **0.97** | **1.54** | **0.0844** | **0.5589** | **0.27** |
|  | *Comamonas* | 6.29 | 0.69 | 57.47 | 0.1032 | 0.5589 | 0.26 |
|  | *Ralstonia* | 6.43 | 0.15 | 268.93 | 0.3286 | 0.8064 | 0.19 |
|  | *Gulbenkiania* | 1.38 | 0.95 | 2.02 | 0.0950 | 0.5589 | 0.27 |
|  | *Tepidiphilus* | 3.45 | 0.99 | 11.98 | 0.0516 | 0.5589 | 0.36 |
|  | *Neisseria* | >999 | <0.001 | >999 | 0.5825 | 0.9125 | 0.20 |
|  | *Citrobacter* | 15.15 | 0.03 | 6836.60 | 0.3834 | 0.8064 | 0.19 |
|  | *Escherichia-Shigella* | 160.10 | 0.37 | >999 | 0.1014 | 0.5589 | 0.33 |
|  | *Serratia* | 27.13 | 0.79 | 935.71 | 0.0677 | 0.5589 | 0.35 |
|  | *Acinetobacter* | 22.69 | <0.001 | >999 | 0.4676 | 0.8571 | 0.22 |
|  | *Enhydrobacter* | 1.58 | 0.12 | 20.01 | 0.7246 | 0.9125 | 0.15 |
|  | *Moraxella* | 0.94 | 0.84 | 1.07 | 0.3661 | 0.8064 | 0.18 |
|  | ***Pseudomonas*** | **1.30** | **1.00** | **1.69** | **0.0532** | **0.5589** | **0.37** |
|  | *Fervidobacterium* | 0.77 | 0.07 | 8.06 | 0.8253 | 0.9125 | 0.14 |
