# Supplementary material for: Nasopharyngeal Bacterial Microbiota Composition and SARS-CoV-2 IgG Antibody Maintenance in Asymptomatic/Paucisymptomatic Subjects
Source: Front Cell Infect Microbiol. 2022 Jul 6;12:882302. doi: 10.3389/fcimb.2022.882302 (PMC9297915; doi:10.3389/fcimb.2022.882302)
Supplement: Supplementary file 11 [file Table_9.docx]

**Consortium UNICORN** (in alphabetical order):

Benedetta Albetti^1^, Claudio Bandi^6^, Tommaso Bellini^7^, Valentina Bollati^1^, Matteo Bonzini^1,8^, Marco Buscaglia^7^, Carlo Cantarella^1^, Laura Cantone^1^, Michele Carugno^1,8^, Sergio Casartelli^9^, Sarah D’Alessandro^10^, Francesca De Chiara^9^, Serena Delbue^10^, Ivano Eberini^11^, Chiara Favero^1^, Luca Ferrari^1^, Monica Ferraroni^12^, Laura Galastri^9^, Cristina Galli^13^, Simona Iodice^1^, Carlo La Vecchia^12^, Chiara Macchi^11^, Alessandro Manenti^14^, Ilaria Manini^3^, Jacopo Mariani^1^, Serena Marchi^3^, Gregorio Paolo Milani^4,5^, Emanuele Montomoli^3^, Elena Pariani^13^, Federica Rota^1^, Angela Cecilia Pesatori^1,8^, Massimiliano Ruscica^11^, Tommaso Schioppo^15^, Letizia Tarantini^1^, Claudia Maria Trombetta^3^, Marco Vicenzi^16,17^, Giuliano Zanchetta^7^

^1^ EPIGET Lab, Department of Clinical Sciences and Community Health, Università degli Studi di Milano, Milan, Italy

^2^ Laboratory of Microbiology and Virology, Department of Life Sciences and Systems Biology, Università degli Studi di Torino, Turin, Italy

^3^ Department of Molecular and Developmental Medicine, Università degli Studi di Siena, Siena, Italy

^4^ Department of Clinical Sciences and Community Health, Università degli Studi di Milano, Milan, Italy

^5^ Pediatric Unit, Fondazione IRCCS Ca’ Granda Ospedale Maggiore Policlinico, Milan, Italy

^6^ Department of Biosciences and Pediatric Clinical Research Center "Romeo and Enrica Invernizzi", University of Milan, Milan, Italy

^7^ Department of Medical Biotechnology and Translational Medicine, University of Milan, Milan, 20129, Italy

^8^ Fondazione IRCCS Ca’ Granda-Ospedale Maggiore Policlinico, Occupational Health Unit, Milan, Italy

^9^ AVIS (Associazione Volontari Italiani Sangue) Milano, Milan, Italy

^10^ Department of Biomedical, Surgical and Dental Sciences, Laboratory of Translational Research, Via Carlo Pascal 36, 20133 Milano, Italy

^11^ Department of Pharmacological and Biomolecular Sciences, University of Milan, Milan, Italy

^12^ Branch of Medical Statistics, Biometry, and Epidemiology "G. A. Maccacaro", Department of Clinical Sciences and Community Health, Università degli Studi di Milano, Milan, Italy

^13^ Department of Biomedical Sciences for Health, University of Milan, Milan, Italy

^14^ VisMederi Research Srl, Siena, Italy

^15^ Division of Rheumatology, ASST Pini-CTO, Milan, Italy

^16^ Fondazione IRCCS Ca’ Granda Ospedale Maggiore Policlinico, Cardiovascular Disease Unit, Internal Medicine Department, Milan, Italy

^17^ Dyspnea Lab, Department of Clinical Sciences and Community Health, University of Milan, Milan, Italy

**UNICORN Consortium Contact: unicorn@unimi.it; Consortium representative: Prof. Valentina Bollati, valentina.bollati@unimi.it**
